# ModBind_dG_: A simulation-based absolute predictor of free energy of binding based on population reweighting

**DOI:** 10.1073/pnas.2513285123

**Published:** 2026-06-15

**Authors:** William Sinko, Blake Mertz, Yoh Terada, S. Roy Kimura

**Affiliations:** ^a^Alivexis, Inc., Cambridge, MA 02142; ^b^Alivexis Inc., Minato-ku, Tokyo 105-0004, Japan

**Keywords:** computational chemistry, molecular dynamics, statistical mechanics, drug discovery, biophysics

## Abstract

Recently, we described ModBind, a powerful simulation-based predictor of ligand off-rates and binding free energy. Here, we describe an update to the method—ModBind_dG_—that allows for the prediction of absolute free energies of binding. ModBind_dG_ is a two-state model for predicting free energy of binding through population-based reweighting of accelerated sampling. We demonstrate theoretically and in practice that ModBind_dG_ can accurately predict the absolute binding free energies of small molecules binding to relevant protein targets. Additionally, we show performance enhancements to the method where ModBind_dG_ can predict up to 2,000× more compounds per day compared to state-of-the-art free energy methods. We show that the method can make accurate predictions on validation datasets as well as active drug discovery programs in our own pipeline. Furthermore, we describe the utility of ModBind_dG_ in a prospective virtual screen that enabled the discovery of multiple chemotypes for a previously “undruggable” protein target. ModBind_dG_ represents an opportunity to significantly impact computational drug discovery, making rigorous physics-based screening of hundreds of thousands to millions of compounds accessible to the entire pharmaceutical community.

## Significance

The absolute binding free energy is a critical determinant of drug efficacy, yet existing computational methods are still too costly to scale for large virtual screening or lead optimization applications. Existing approaches rely on alchemical transformations, empirical approximations, or path-biased sampling to solve the fundamental issue that at physiological temperatures, unbinding events are too rare to routinely observe within simulation timescales. Derived from statistical mechanics, ModBind_dG_ utilizes path-independent, high-temperature sampling to enable rare transitions, combined with population reweighting to recover rigorous thermodynamics. Validated on hundreds of ligand–protein complexes for diverse targets, ModBind_dG_ matches the accuracy of industry-leading methods while enabling orders of magnitude higher throughput, enabling large-scale physics-based screening of drug candidates prior to experimental synthesis and testing.

The drug discovery process is often slow and expensive with great effort expended in each of the hit identification, hit-to-lead, and lead optimization steps. Computational chemistry aims to accelerate the process and reduce costs by identifying promising drug candidates and guiding synthetic decisions of medicinal chemists. A critical tool in that computational workflow is molecular dynamics (MD) simulations. MD has always been appreciated for its rigor and potential accuracy, but only recently has it become practical to use MD simulations in drug discovery due to the advent of fast scalable hardware like graphics processing units (GPUs) and significant improvements in workflow and methodology (1, 2). Relative free energy predictions [e.g., free energy perturbation (FEP) (2, 3) and thermodynamic integration (TI) (4)] are the most notable MD approaches, but their strength lies in the evaluation of closely related compounds such as those found in lead optimization programs. Numerous other methods exist to characterize small molecule–protein interactions including steered MD (5, 6), metadynamics, (7, 8) targeted MD (9), ligand Gaussian accelerated MD (10–12), random accelerated MD (13, 14), and weighted ensemble methods (15–17). These methods are all valuable tools to the computational chemist; however, each of them suffers from some combination of a lack of extensive validation (hundreds of ligands, multiple targets), high computational expense, required domain expertise, and inconsistent performance with respect to absolute free energy predictions. Recently we (18) and others (19) have proposed methods to approximate absolute binding free energy as a means to enrich diverse chemical library screens for hit finding, a practical impossibility until now.

We originally developed scaled MD as a rigorous methodology to calculate free energy landscapes of biomolecular conformational sampling (20). Mollica et al. developed an extension to the theory which allowed for the calculation of relative off-rates among various ligand series (21, 22). More recently we reported on the development of ModBind, a scaled MD approach capable of predicting accurate ligand binding off-rates. We demonstrated that these off-rates are a reasonable substitute for free energy rankings and can be calculated at unprecedented speed (hundreds of compounds/day/GPU) (18). ModBind showed good correlation across 10 different targets and hundreds of ligands for both off-rate and free energy prediction. While the original population-based reweighting method was theoretically capable of predicting binding energies directly, it was impractical for drug discovery applications: The simulations would have to be extremely long (>μs) to capture enough unbinding and binding events to be statistically accurate. For this reason, we derived a new theory to use a two-state model with population-based reweighting, effectively combining the accelerated sampling of scaled MD with the rapid convergence of population-based methods (18, 20). The method is in principal similar to end-state free energy approaches (23) but with the unique advantage of computing the ratio of populations to derive the binding energy. Our theory adds a distinct methodology to the free energy prediction field and is simple and efficient. We extensively validate our theory across multiple drug discovery targets and hundreds of ligands as well as showing its potential in prospective use cases.

## Theory.

Our method, ModBind_dG_, computes absolute binding free energies from the equilibrium ratio of bound and unbound populations. It is a two-state model consisting of two MD simulations: 1) the ligand bound to the protein in water (the bound state), and 2) the ligand in bulk solvent (the unbound state). High-temperature MD simulations significantly increase the rate of sampling and escape events to an absorbing boundary between the bound and unbound states. We ensure flux balance between the states to maintain detailed balance. Configurations are binned along a reduced coordinate system describing the dominant configurational change, and bin populations are reweighted to 300 K using population-based reweighting. From the reweighted bins we recover the free energy distribution of the two states at room temperature and their corresponding populations under flux balance. Summation of the population bins composing each state yields the population ratio, allowing us to calculate the standard binding free energy via the Helmholtz free energy equation.

In principle, our method follows the logic of brute-force equilibrium MD, in which state populations are counted directly from a single long trajectory sampling both states. Such simulations are impractical or impossible for most drug binding events given their characteristic timescales and current limitations of computational hardware. We overcome this obstacle by accelerating sampling with high-temperature MD and population reweighting. Framing our system as a two-state model with absorbing boundaries further accelerates convergence of population ratios. These methodology design choices are grounded in statistical mechanics and validated by empirical testing, as shown below.

The probability density of a phase-space point is given by

[1] $$P\left(x\right)=\frac{e^{-\beta H\left(x\right)}}{Q}\text{where}\;\beta =\frac{1}{k_{B}T},$$

with $k_{B}$ as the Boltzmann constant, *H* is the Hamiltonian as a function of *x* (the momenta and coordinates), and *Q* is defined as the canonical partition function:

[2] $$Q=\frac{1}{h^{3N}\prod_{\alpha }N_{\alpha }!}\int e^{-\beta H\left(x\right)}dx,$$

where h is Planck’s constant raised to the power of *3N*, and *N* is the number of particles. $\Pi_{\alpha }N_{\alpha }!$ accounts for indistinguishable particles where $N_{\alpha }$ is the number of particles within each species α (24) *Q* can rarely be solved analytically for complex systems. However, the momentum integrals and indistinguishability factors in *Q* are common to both states (e.g., bound and unbound), and *Q* cancels in the ratio of populations for free energy differences between states (24, 25). We therefore work directly from the configurational integrals of the non-normalized populations $p\left(\vec{r}\right)$, which may be used in free energy calculations when the partition function is not practical to solve:

[3] $$p\left(\vec{r}\right)=e^{-\beta V\left(\vec{r}\right)}\approx C\frac{t_{\;{\vec{r}}_{i}}}{\Delta t},$$

where $V\left(\vec{r}\right)$ is the potential energy of the coordinates of $\left(\vec{r}\right)$, $t_{\;{\vec{r}}_{i}}$ is the time spent in configuration(s) ${\vec{r}}_{i}$ (related configurations in bin i), and Δt is the frame capture interval. *C* is a proportionality constant that allows the conversion between populations of simulations of finite length to energy on a potential energy surface. *C* will cancel in any ratios of populations and does not need to be solved for the free energy difference calculations described here. Use of the notation ${\vec{r}}_{i}$ refers to bins of related configurations, and $\vec{r}$ refers directly to individual configurations in this manuscript. This is because $p\left(\vec{r}\right)$ may be evaluated from each distinct configuration based on the energy of the configuration for simple systems. However, for complex systems, and because configurational space is continuous, it is standard practice in the MD field to approximate $p\left(\vec{r}\right)$ as $p\left({\vec{r}}_{i}\right)$, the time spent in the configuration(s) of bin *i* over a simulation of finite length. The approximate equality becomes exact as the bin volume of *i* goes to zero and the length of the simulation goes to infinity, fulfilling the ergodic hypothesis. Importantly, Eq. (**3**) establishes the relationship between configurational time, simulation parameters (i.e., frame capture interval), and the corresponding potential energy. The system will spend more time in lower-energy configurations compared to higher-energy ones under any simulation parameters. For a system at different temperatures or on a scaled potential where a constant λ alters the temperature or the potential energy function $e^{-\beta V\lambda \left(\vec{r}\right)}$, the modified populations of individual configurations $p^{*}\left(\vec{r}\right)$ can be corrected to the canonical populations $p\left(\vec{r}\right)$ by raising to the power of 1/λ (20):

[4] $$p\left(\vec{r}\right)=p^{*}{\left(\vec{r}\right)}^{1/\lambda }.$$

This relationship allows us to relate free energy differences along any coordinate by integrating along a modified potential; more practically for complex systems, this means simulating on a biased potential or temperature and counting binned populations $p^{*}\left({\vec{r}}_{i}\right)$ in bins of equal volume. The counts may then be corrected for *λ*—which is equivalent to the temperature scaling factor T/T^*^—yielding $p\left({\vec{r}}_{i}\right)$. We can extend this formalism to include multiple states of a system by considering ratios of populations defined by Eq. **4** (assuming the system is at equilibrium or quasi-equilibrium) as demonstrated in Sinko et al. (20) Conceptually our method is similar to hyperdynamics (26, 27) or accelerated MD (28) and other methods that apply a boost potential to all or part of the potential energy surface. However, reweighted ratios of populations related to Eq. **4** converge more rapidly than reweighting based on an energetic boost potential (20). Although it is impractical to fully describe the high-dimensional space of folded biomolecules, this is generally not required as biological targets act as systems with substantially reduced dimensionality (29, 30). We and others have proposed that properties of interest, in particular the free energy of states, can be well-approximated by binning along low-dimensional space of a given system (18, 20, 21, 31, 32).

The ratio of populations of states is proportional to the probability of transitioning between states when detailed balance is maintained. If $P_{\mathrm{AB}}$ is the probability of transitioning from A to B in a fixed time interval and $P_{\mathrm{BA}}$ is the probability of transitioning from B to A in the same fixed time interval, given detailed balance:

[5] $$\left(\int_{A}e^{-\beta V\left(\vec{r}\right)}d\vec{r}\right)\times P_{\mathrm{AB}}=\left(\int_{B}e^{-\beta V\left(\vec{r}\right)}d\vec{r}\right)\times P_{\mathrm{BA}}.$$

Equilibrium is achieved when flux between states is equal. Rearranging gives us the relative population ratio based only on the ratio between transition probabilities:

[6] $$\frac{\int_{A}e^{-\beta V\left(\vec{r}\right)}d\vec{r}}{\int_{B}e^{-\beta V\left(\vec{r}\right)}d\vec{r}}=\frac{P_{\mathrm{BA}}}{P_{\mathrm{AB}}}.$$

The inverse relationship between the integrated populations of a state and the transition probability out of that state is critical to understanding our approach. When transitions happen rapidly, populations of the state are small.

**Fig. 1.**
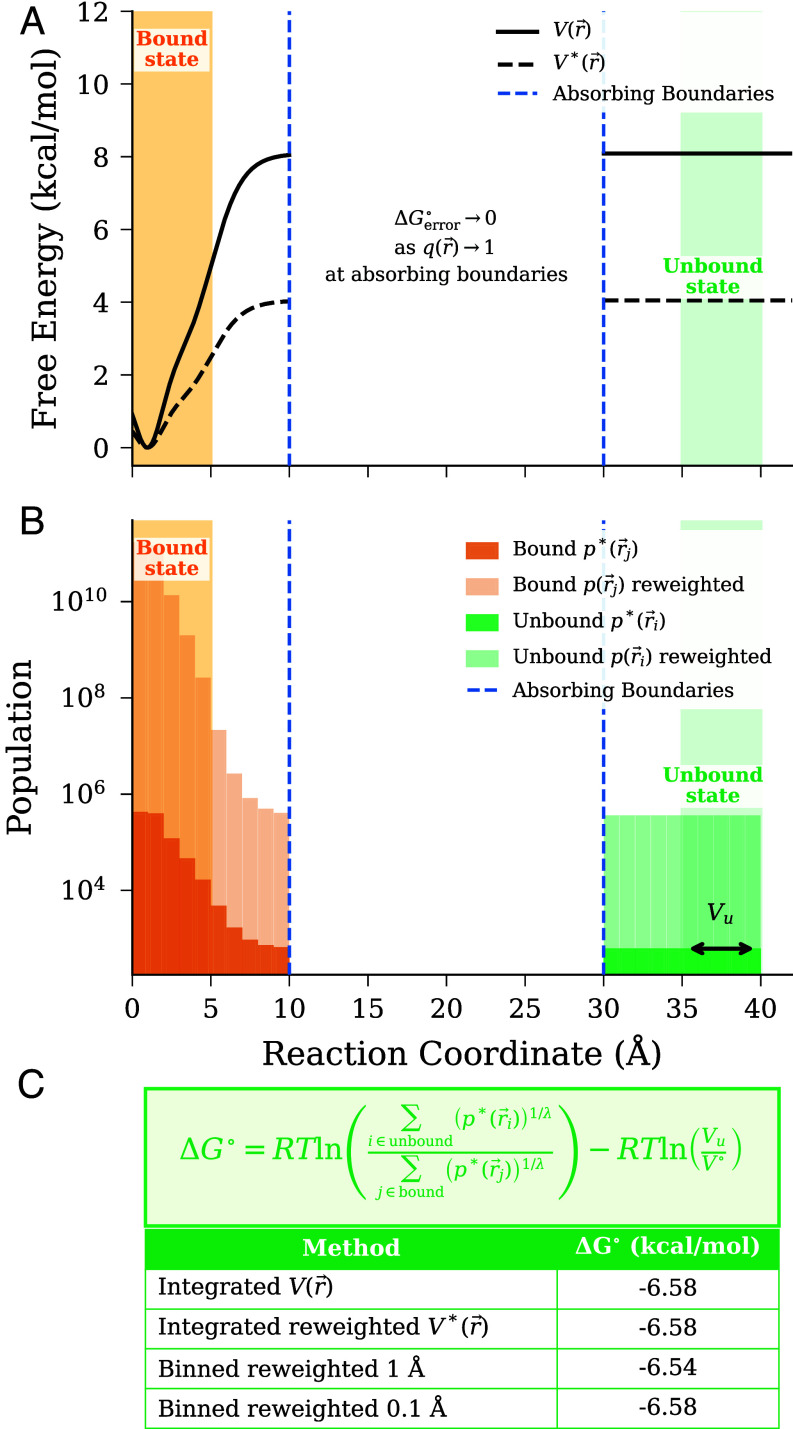
Overview of the ModBind_dG_ method. (*A*) Simulate the bound and unbound states on an accelerated potential until escape to an absorbing surface. Shown are the potential of mean force (PMF) profiles for the unbiased [*V*$\left(\vec{r}\right)$ (*solid*)] and the accelerated potential [V*$\left(\vec{r}\right)$ (*dashed*)] along the reaction coordinate. *Orange and green shading*: bound and unbound state regions, respectively. *Blue dashed lines*: absorbing boundaries. (*B*) Count, bin, and reweight populations along the relevant reaction coordinate. Population histograms are shown for both the bound (*orange*) and unbound (*green*) states and were computed from using the hypothetical accelerated potential V*$\left(\vec{r}\right)$ in 1*A*. *Solid bars*: populations p*$\left(\vec{r}\right)$ sampled at high temperature; *translucent bars*: reweighted populations *p*$\left(\vec{r}\right)$, obtained via Eq. **4**. *V*_u_: volume of the unbound state used to correct to standard state. (*C*) The standard binding free energy Δ*G*° is computed from Eq. **14**. The table reports Δ*G*° values computed from the hypothetical 1D energy surface and state definitions, in Fig. 1*A*, by four approaches: direct integration of *V*$\left(\vec{r}\right)$ the ground truth answer, integrated reweighting from V*$\left(\vec{r}\right)$ (exact), and binned reweighting at 1 Å (from Fig. 1*B*) and 0.1 Å resolution. The agreement between direct and reweighted values (−6.58 kcal/mol) validates the reweighting procedure under ideal conditions; the small deviation at 1 Å bin resolution (−6.54 kcal/mol) illustrates the effect of well-known bin discretization error in our Riemann sum, which vanishes at finer resolution.

One way to obtain the free energy difference between two states is to sample the system at equilibrium, observe many transitions between the two states, and quantify the probability of each respective state. The Helmholtz free energy difference between states A and B is expressed in the analytical form as an integral over the phase space volumes of A and B across the potential energy landscape $V\left(\vec{r}\right)$:

[7] $$\Delta F_{A\to B}=-RT\text{ln}\left(\frac{\int_{A}e^{-\beta V\left(\vec{r}\right)}d\;\;\vec{r}}{\int_{B}e^{-\beta V\left(\vec{r}\right)}d\;\;\vec{r}}\right).$$

(The *NVT* ensemble yields ΔF, but most drug discovery experiments are conducted in the *NPT* ensemble which yields the Gibbs free energy Δ*G*. Because the pressure–volume change in drug binding is negligible (31), we can assume ΔG≈ΔF. We will use ΔG notation to be consistent with experiments and general terminology going forward.) If we assume that the dynamics of a system that contains states A and B are Markovian, decoupled from previously sampled states, and can only move to other states, then it is possible to separately sample states A and B until a sufficient number of escape events are observed. An escape event is defined as movement of sufficient distance away from the original state such that there is a low probability of returning to that state. This condition does not require an irreversible process; rather an escape event can be defined as movement to an absorbing boundary. To meet the requirements of detailed balance, the number of transitions from state A to state B and from B to A must be equal (i.e., equal flux). Thus, Eq. **7** may be calculated from a long MD simulation observing transitions between states (33), or it may be calculated with multiple simulations observing an equivalent number of escapes to an absorbing boundary between both states. In the latter case, the energy level at the absorbing boundary should be nearly equivalent between states A and B to avoid an unbalanced accounting between states. Furthermore, we must ensure there is little chance of return to the starting state (with meaningful contribution to the population). This approach provides an exact solution when the absorbing boundaries between states A and B are coupled (i.e., there is no volume between them). However, in the condition under which the absorbing boundaries have a nonzero volume between them, the probability $q\left(\vec{r}\right)$ at the point of the absorbing boundary (probability the configuration that is absorbed is committed to changing states) indicates high probability of transition to the next state. $q\left(\vec{r}\right)$ is defined analogously to a general committor function:

[8] $$q\left(\vec{r}\right)=\frac{n_{B}\left(\vec{r}\right)}{n_{A}\left(\vec{r}\right)+n_{B}\left(\vec{r}\right)},$$

where $n_{B}\left(\vec{r}\right)$ is the number of trajectories initiated from configuration $\left(\vec{r}\right)$ that reach state B before state A and $n_{A}\left(\vec{r}\right)$ is the number of trajectories reaching state A first. Here, $q\left(\vec{r}\right)$ is defined as a general committor function which gives the probability of forward state transition at any given placement of an absorbing boundary. We define two committors in two independent reference frames and for two absorbing boundary positions: one for trajectories initiated in the bound state and another for trajectories initiated in the unbound state. While Fig. 1 displays both states on a unified PMF for clarity, the underlying simulations use independent coordinate systems originating at each starting state, and independent committor functions may be calculated. Any error introduced by the volume between absorbing boundaries approaches zero as $q\left(\vec{r}\right)\to 1$ at the state’s respective absorbing boundary position. When $q\left(\vec{r}\right)∼1$, (i.e., high probability of state transition), we can obtain Δ*G*_AB_ by computing the populations of each respective state without sampling the region between absorbing boundaries. Additional contributions to a state’s population occur only when a simulation returns to the state after leaving it. We demonstrate theoretically and empirically even at moderate $q\left(\vec{r}\right)\approx 0.5$ or above that the effect on population ratios for binding free energy is minimal, but the simulation efficiency is greatly improved by including absorbing boundaries with some volume between them (*Results and Discussion* and *SI Appendix*).

We can now begin to formulate the free energy relationship based on discrete MD simulations of the two distinct states using Eqs. **6** and **7**. Since we are only concerned with states A and B, it is not necessary to integrate the entire continuous energy surface of the partition function Q. The approach of using two separate states to characterize the free energy surface can be applied to MD simulations of ligand unbinding so long as flux balance is maintained. We show below that this is indeed the case, which unlocks significant speedups to the computation of free energy differences between states. Now the free energy difference between state A and state B is given by

[9] $$\begin{array}{c} \Delta G_{A\to B}=-RT\text{ln}\left(\frac{\int_{A}e^{-\beta V\left(\vec{r}\right)}d\;\;\vec{r}}{\int_{B}e^{-\beta V\left(\vec{r}\right)}d\;\;\vec{r}}\right)≅-RT\text{ln}\left(\frac{P_{B\to }}{P_{A\to }}\right), \end{array}$$

where $P_{A\to }$ and $P_{B\to }$ is the probability that state A and state B have transitioned to the absorbing boundary. As $q\left(\vec{r}\right)\to 1$ in both directions, detailed balance is achieved, and we can directly substitute $P_{A\to }$ for $P_{\mathrm{AB}}$ and $P_{B\to }$ for $P_{\mathrm{BA}}$ into our relationship for detailed balance in Eq. **5**. In a two-state model under detailed balance and flux balance, one must simulate the same number of escapes from state A as from state B. Assuming $P_{A\to }≅P_{\mathrm{AB}}$, and $P_{B\to }≅P_{\mathrm{BA}}$, all that is required is to define the absorbing boundary to ensure a low probability of returning to the original state.

To properly characterize the energy landscape of a transition process, one should start simulations from a Boltzmann-weighted distribution along the free energy surface. In practice one may start the simulations from any point in state A or state B, assuming that each state is stable enough for adequate sampling of the respective energy basin, and that escape from these states occurs in a quasi-Markovian manner. This behavior fulfills approximate symmetry of the system and is characteristic of the equilibrium surface-based feedback loop model proposed by Bhatt and Zuckerman (34). In the case of ligand binding, transitions from the bound state are driven by enthalpic contributions that are characteristic of a single deep energy well, whereas transitions from the unbound state are more entropically driven due to the flat energy surface. Starting simulations from the bound state, where $V\left(\vec{r}\right)$ is most favorable (e.g., the crystallographic or docked pose), and from an arbitrary position in a volume along the unbound state where $V\left(\vec{r}\right)$ is constant (e.g., the next frame after crossing an absorbing boundary), provide a good approximation of starting from a Boltzmann-weighted distribution. The ligand binding/unbinding process is especially suitable to this approach because we may define the absorbing boundaries between bound and unbound when the potential is nearly flat, and the integrated volume of the unbound state $V_{u}$ is arbitrary as long as it can be corrected back to standard state free energy.

Now that we have a theoretical basis for our two-state model, we can relate ModBind_dG_ to experimental measurements. The Gibbs free energy of binding for a ligand is a function of the equilibrium dissociation constant (*K*_d_), the gas constant (*R*), temperature (*T*), and standard concentration $C^{{}^{\circ}}=\frac{1}{V^{{}^{\circ}}}$ (The standard state $C^{{}^{\circ}}=1M$ implies that $V^{{}^{\circ}}$ = 1,661 Å^3^):

[10] $$\Delta G^{{}^{\circ}}=RT\text{ln}\left(\frac{K_{d}}{C^{{}^{\circ}}}\right).$$

In our free energy prediction at standard state concentration, we use the standard state free energy of binding from Doudou et al. (35) Buch et al. (36) and more generally shown by General et al. (37):

[11] $$\Delta G^{{}^{\circ}}=\Delta G_{comp}-RT\text{ln}\left(\frac{V_{u}}{V^{{}^{\circ}}}\right),$$

where

ΔGcomp=RTln∫unbounde-βV r→d  r→∫bounde-βV r→d  r→.

is the computed free energy difference between the bound and unbound states and *V*_u_ is the effective volume of the unbound state. The term $-RT\text{ln}\left(\frac{V_{u}}{V^{{}^{\circ}}}\right)$ accounts for the free energy difference of changing from the standard state volume to $V_{u}$, the unbound simulation volume. The choice of $V_{u}$ is defined by the limits of integration for the unbound term where $e^{-\beta V\left(\vec{r}\right)}$ is constant. Varying $V_{u}$ should not affect the calculation of $\Delta G^{{}^{\circ}}$ beyond statistical uncertainty (36, 37). Thus, our general computed free energy is given by

[12] $$\begin{array}{c} \Delta G^{{}^{\circ}}=RT\text{ln}\left(\frac{\int_{\text{unbound}}e^{-\beta V\left(\vec{r}\right)}d\;\;\vec{r}}{\int_{\text{bound}}e^{-\beta V\left(\vec{r}\right)}d\;\;\vec{r}}\right)-RT\text{ln}\left(\frac{V_{u}}{V^{{}^{\circ}}}\right), \end{array}$$

and is further adapted to the case when those simulations must be reweighted after running on a scaled potential to accelerate unbinding:

[13] $$\begin{array}{c} \Delta G^{{}^{\circ}}=RT\text{ln}\left(\frac{\int_{\text{unbound}}{\left(e^{-\beta \lambda V\left(\vec{r}\right)}\right)}^{1/\lambda }d\;\;\vec{r}}{\int_{\text{bound}}{\left(e^{-\beta \lambda V\left(\vec{r}\right)}\right)}^{1/\lambda }d\;\;\vec{r}}\right)-RT\text{ln}\left(\frac{V_{u}}{V^{{}^{\circ}}}\right), \end{array}$$

We note however that λ cancels and Eq. **13** simplifies to Eq. **12** after reweighting. Considering a discrete MD simulation, one would estimate the integral by summing reweighted configurations in the bound and unbound state, where *i* iterates over all bins of the unbound state and *j* over the bound state. Here, we describe $\Delta G^{{}^{\circ}}$ in terms of a discrete MD simulation that has been scaled by λ:

[14] $$\Delta G^{{}^{\circ}}=RT\text{ln}\left(\frac{\sum_{i\in unbound}{\left(p^{*}\left(\vec{r_{i}}\right)\right)}^{1/\lambda }}{\sum_{j\in bound}{\left(p^{*}\left(\vec{r_{j}}\right)\right)}^{1/\lambda }}\right)-RT\text{ln}\left(\frac{V_{u}}{V^{{}^{\circ}}}\right).$$

Eq. **14** is used for the calculation of all $\Delta G^{{}^{\circ}}$ of binding in this manuscript. Using the relationship in Eq. **14**, if two independent simulations describe the populations of the endpoints (bound and unbound) along a reaction coordinate (e.g., distance between the center of mass (COM) of a ligand and the target molecule), we can estimate the free energy of binding using any scaled potential or temperature. Flux balance must be maintained when applying Eq. **14**, so that the ensemble maintains detailed balance, and this is achieved by running an equal number of simulations to escape between bound and unbound states.

For complex systems, one must reduce the dimensionality of the system to the most important degrees of freedom to make the problem computationally tractable. This means that *i* and *j* are treated as finite bins, making the reweighting approximate. As long as the choice of reduced dimensions are sufficient to fully describe the slow motions of the system (and the nondescribed dimensions contribute a constant entropy to the states), this is a reasonable approximation (20, 31, 32).

Using the absorbing boundaries formalism, our method essentially measures the average escape time from states under accelerated conditions (high temperature or scaled potentials) through counting and binning populations along a coordinate system. Reweighting produces the canonical population ratios, which are proportional to the canonical probabilities of transition between states. Greater populations are observed in states with more favorable free energies because the escape rate is slower, i.e., more time is spent in that state. It is unnecessary to simulate the entire partition function or the ligand unbinding pathway; it is sufficient to find reasonably converged estimates of the ratio of the bound and unbound states by modeling each system until it reaches the absorbing boundaries.

Here, we provide a schematic workflow of ModBind_dG_. First, we simulate at high temperature or on an accelerated potential, $V^{*}\left(\vec{r}\right)$, separated by absorbing boundaries chosen so that the probability of transition between states $q\left(\vec{r}\right)$ is estimated to be high (Fig. 1*A*). The result holds exactly as $q\left(\vec{r}\right)$ → 1. Next, we count and bin populations generated under accelerated conditions; generally a reduced dimensional space describing the most important degrees of freedom is used. Bins are then reweighted by Eq. **4** to achieve the populations at room temperature (Fig. 1*B*). Flux balance is ensured by simulating an equal number of bound and unbound simulations. The volume of the unbound state ($V_{u}$) is used to correct to standard state concentration. Next, we utilize Eq. **14** to calculate the free energy difference; either the integral of $V\left(\vec{r}\right)$ or reweighted integral of $V^{*}\left(\vec{r}\right)$ provide an exact answer for $\Delta G^{{}^{\circ}}$. The binning and reweighting procedure matches closely and is exact as the bin size approaches zero (Fig. 1*C*). ModBind_dG_ directly implements this workflow through explicit separate simulations of a two-state system (i.e., ligand in solution and ligand–target complex) using population reweighting theory and acceleration via a scaled potential.

As an initial proof of this concept for the two-state model, we constructed a simple two-well system along a 1-D reaction coordinate with an arbitrary energy difference between the two wells (*SI Appendix*, Fig. S1). Two conditions were tested: 1) long equilibrium simulations between the two wells (continuous MD) and 2) simulations started from the base of either well 1 or well 2 and repeatedly restarted at the initial configuration when the system reached the peak of the barrier separating the two wells (discrete state MD). 10,000 simulations were conducted for each condition to ensure convergence. We calculated the underlying populations of microstates by binning along the 1-D reaction coordinate. The ratio between the most-occupied bins of well 1 versus well 2 provide the free energy difference between the two states. Simulation of this simple 1D system resulted in nearly identical free energy estimates for both the continuous and discrete cases (*SI Appendix*, Fig. S1 *D*–*F*). However, the discrete system converged by a factor of 10X faster than the continuous system (*SI Appendix*, Fig. S2). This indicates that our method of using a two-state system to sample populations of bound and unbound ligands (discrete state MD—i.e., ModBind_dG_) is much more efficient than using a single long simulation to capture multiple crossing events between the energy wells (continuous MD). Details of the 1-D model system can be found in the *SI Appendix*.

It is important to note that the number of escape events must be equal between states A and B to maintain detailed balance in discrete state MD; it is also acceptable to normalize the populations of each state if different numbers of escape simulations are performed for either state. In the two-state model, this means that the total population count for each state is dependent upon 1) the total number of simulations performed until escape and 2) the frame capture rate of the simulation. The individual raw populations of the bound and unbound states in the two-state model are proportional to the free energy of the state, but not equal to that free energy since they depend upon simulation parameters. However, the ratio between the populations of the two states can be directly related to the free energy difference between the states and are independent of simulation parameters such as number of simulations and frame capture rate; these cancel out in the ratio for Eqs. **12**–**14**. It is important to note that the unbound term of Eq. **14** is not a free energy of solvation, but rather a measure of the stochastic occupancy of a molecule in a reference volume which is directly proportional to the diffusion rate of the ligand in solution. Therefore, it is possible to provide a reasonable estimate of the population of the ligand in the unbound state using the Stokes–Einstein–Sutherland equation (see *SI Appendix* for details).

The accuracy of population reweighting calculations requires sufficient sampling of the states, proper definition of the bound state, and generally higher frame capture rates than other MD methods so that the large population differences can be well-approximated, especially with higher energy states included in the unbound-to-bound ratio (20, 32). Although one might argue that it should include the entire unbinding pathway or potential intermediate states, FEP and related alchemical methods provide empirical evidence supporting the use of only sampling the tightly bound state: They obtain good agreement with experimental results by simply sampling ligand movement around a local minimum. This suggests that the binding free energy is often dominated by a tightly bound state and that characterization of the loosely bound state (e.g., >2 Å from the protein binding pocket) plays a minor role in the binding free energy for most ligand–protein interactions in drug discovery. Thus, we expect that characterization of the tightly bound state is sufficient in most cases to capture the thermodynamics of the bound state.

## Materials and Methods

Protein structures originated from the PDB and were prepared for modeling with Schrödinger Protein Preparation Wizard (38). A predicted binding pose was required as the starting structure for all MD simulations; ligand posing was performed with Glide (39, 40) or Autodock Vina (41) using structural guidance (maximum common substructure overlay to the ligand in the crystal structure), when possible. After minimization and equilibration, MD simulations were conducted at high temperatures, 550 to 1,200 K, maintained via the Langevin thermostat in the NVT ensemble with 2 fs timestep using openMM (42). Force field parameters were taken from ff14SB (43) (protein), TIP3P (44) (water model), and the General Amber Force Field 2.1 (45) generated via the openmmforcefields (46) package (ligand). The bound state simulations contained the ligand–protein complex, and the unbound state contained only the ligand, both were solvated in a box of water. In the bound state restraints were applied to the backbone atoms of the protein more than 6 Å from the ligand to maintain secondary structure at high temperatures.

MD simulations of the ligand in the bound state and alone in bulk solvent were performed until an escape event occurred and the simulation was absorbed at the absorbing boundary; multiple independent replicas with flux balance maintained (i.e., equal numbers of bound and unbound simulations) reduced statistical uncertainty. From the simulations, we computed the ligand positional movements using center of mass (COM) Cartesian coordinates from the starting position referenced to the protein structure. We summed the frame count in each Cartesian cubic bin (maintaining equal volumes), and recovered populations at 300 K by reweighting with Eq. **4**. The reweighted population ratio of states was computed from the sum of reweighted bin populations in each state. In the two-state system, the bound state was defined by 0 to 2 Å COM movement from the starting position, and the unbound state was defined by 0 to 5 Å COM movement from the starting position, with the absorbing boundary placed at 5 Å COM for both states. The state definitions are measured in radial distance from the starting position, and the reweighting uses cubic bins to maintain equal phase space volume among bins. Binding free energies were computed from the absolute reweighted population ratios of the bound and unbound state after ensuring flux balance using Eq. **14**. Uncertainty was quantified via bootstrapping to compute a 95% CI. The ModBind_dG_ core code (MD simulation and analysis) was written in python without AI assistance. Claude Opus 4.5 and 4.6 were used in part to assist code writing for the following: secondary statistical analysis, visualizations, process automation, and quality checks on scientific rigor. The authors verified the contents of all AI-generated outputs. Generally, we follow the best practices for quantifying uncertainty and sampling as described by Grossfield et al. (47) and for calculating free energy of binding and evaluating performance as described in Hahn et al. (48) Comprehensive details of the methodology used in this work is provided in the *SI Appendix*.

## Results and Discussion

### Validation of ModBind_dG_.

**Fig. 2.**
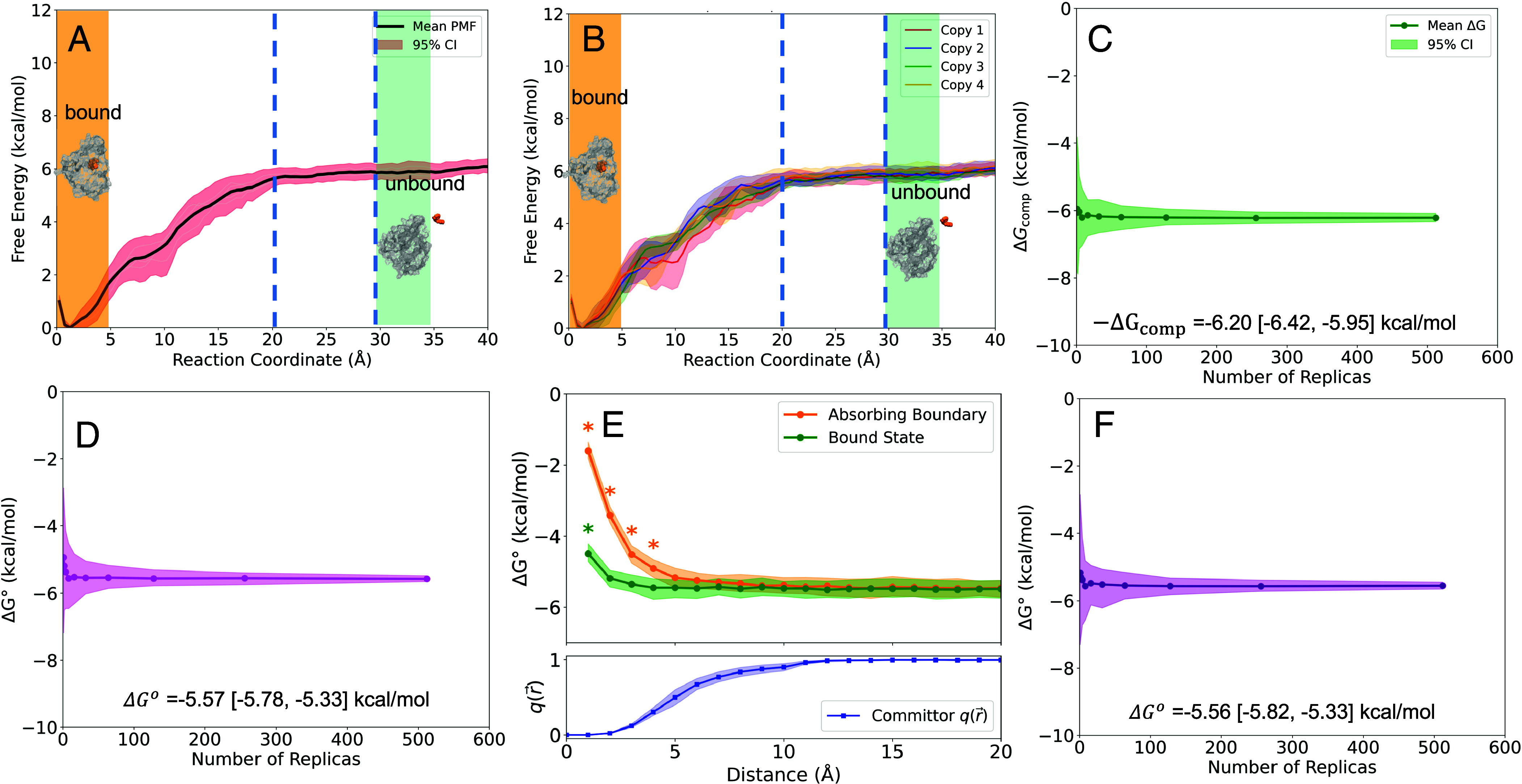
Free energy of high temperature MD simulations of unbinding can be approximated for benzamidine trypsin. (*A*) Potential of mean force (PMF) from high temperature scaled MD simulation reweighted via population-based reweighting. Reaction coordinate is defined as the change in the distance of the center of mass (COM) of the ligand from the initial position in the bound state, 128 individual replicas were used to calculate a mean and 95% CI, bootstrapped from a pool of 512 replicas. (*B*) PMF of benzamidine unbinding from trypsin using four unique groups of 128 replicas. The profile of the PMF is highly consistent across copies. (*C*) Convergence of the ${\Delta G}_{comp}$ from Eq. **11**, the number of replicas required to obtain a converged final result of the full dataset of 512 replicas is relatively low. (*D*) Convergence of the standard state free energy $\Delta G^{{}^{\circ}}$ from the full PMF as obtained from Eq. **14** as a function of the number of simulations. (*E*) Convergence of $\Delta G^{{}^{\circ}}$ as a function of the position of the bound state boundary condition of the absorbing boundary (Orange) or the bound state distance from the starting position is changed (green) * indicates statistically significant difference *P* < 0.05 from maximum distance (IE convergence not reached). The probability $q\left(\vec{r}\right)$ is plotted in the lower panel indicating the probability of reaching the unbound state prior to the bound state at each distance. (*F*) Convergence of the standard state free energy $\Delta G^{{}^{\circ}}$ as obtained from Eq. **14** after utilizing absorbing boundaries at 20 and 30 Å from which trajectory information is deleted between the boundaries. For all plots the lightly shaded regions represent the 95% CI of the mean, and the dark solid line is the mean value based on bootstrapping with replacement.

In order to validate the proposed two-state approach of ModBind_dG_ for a protein–ligand system, we initially modeled benzamidine bound to trypsin (49). This system has been studied extensively to understand the structure–function relationship and the free energy of binding (50, 51). By allowing the ligand to diffuse from the bound state (0 to 5 Å) to the unbound state (~30 to 35 Å) we can count the populations of conformations in a uniform Cartesian grid and reweight these populations (Eq. **4**). The potential of mean force (PMF) can be obtained by summing our populations along the reaction coordinate (COM distance) and normalizing to zero at the minimum (Fig. 2 *A* and *B*). Increasing the number of replicas in our PMF calculations does not change the overall results for our computed free energy ${\Delta G}_{comp}$ = −6.20 [−6.42, −5.95] kcal/mol (Fig. 2*C*), and our standard state free energy $\Delta G^{\circ }$ = −5.57 [−5.78, −5.33] kcal/mol (Fig. 2*D* and *SI Appendix*, Fig. S3*A*) agrees well with both experiment (−6.4 kcal/mol) and previous free energy calculations using umbrella sampling (−7.3 kcal/mol) (35), brute force simulations (−5.2 kcal/mol) (36), and On-the-fly Probability Enhanced Sampling (−6.4 kcal/mol) (52).

The next step was to determine the trade-off between accuracy and efficiency of ModBind_dG_. Although the mean $\Delta G^{\circ }$ from bootstrapping was accurate in as little as 1 replica (~3 ns total simulation time) we noted significant statistical variance that decreased to acceptable levels of less than 1 log order 95% CI at 8 to 16 replicas (~25 to 50 ns total simulation time). We then determined the minimum bound state size and starting position of the absorbing boundary without significantly altering the calculated $\Delta G^{{}^{\circ}}$ (Fig. 2*E*). Effectively we can assign the absorbing boundary at ≥5 Å with minimal effect on the $\Delta G^{{}^{\circ}}$. This is corroborated by $q\left(\vec{r}\right)$ in the bound state, where $q\left(\vec{r}\right)$ is the probability of immediately reaching the unbound state, at each position along the reaction coordinate. Even at moderate $q\left(\vec{r}\right)$, where the probability of rebinding and unbinding are approximately equal, at 5 Å COM movement, the $\Delta G_{\mathrm{error}}^{o}$ associated with the absorbing boundaries volume is only ~0.3 kcal/mol and no longer statistically significant. Additionally, we provide analysis of $q\left(\vec{r}\right)$ in the unbound state simulations where, $q\left(\vec{r}\right)$ represents the probability of the ligand leaving the unbound states defined volume and not returning under simulation conditions. This conclusion is additionally justified by simple Markov-state modeling (*SI Appendix*). Furthermore, simulations in each state are both subject to absorbing-boundary-driven $\Delta G_{\mathrm{error}}^{o}$ of comparable magnitude (at distances ≥5 Å); in Eq. **14** these errors enter as population count errors in both the numerator and denominator and largely cancel, yielding a residual error in $\Delta G^{\circ }$ that is significantly smaller than the individual state component errors.

Additionally, we tested the sensitivity of $\Delta G^{\circ }$ to the bound state definition and found minimal change after 2 Å COM distance. This result agrees with our hypothesis that most ligand–protein binding is driven by a deep energetic well, and loosely bound states ≥2 Å COM have minimal contributions to the bound state. To test this hypothesis, we assigned an absorbing boundary between 20 to 30 Å COM distance by removing the corresponding portion of the simulation. Our standard free energy $\Delta G^{\circ }$ = −5.56 [−5.82, −5.33] kcal/mol (Fig. 2*F*) for simulations with absorbing boundaries is in excellent agreement to our results without absorbing boundaries $\Delta G^{\circ }$ = −5.57 [−5.78, −5.33] kcal/mol (Fig. 2*D*). Even with a larger distance between absorbing boundaries (10 to 30 Å COM) there was little change to $\Delta G^{\circ }=-5.48\left[-6.75,-5.24\right]$ (*SI Appendix*, Fig. S3*B*). This unambiguously demonstrates that inserting absorbing boundaries at high $q\left(\vec{r}\right)$ has a negligible effect on the calculated $\Delta G^{\circ }$.

Next, we tested whether we could utilize a true two-state system, with 1) a bound state where the ligand unbinds until reaching the absorbing boundary and 2) a separate simulation of the ligand in bulk solvent for the unbound state. Utilizing the two-state system, we calculated $\Delta G^{\circ }$ = −5.73 [−6.13, −5.49] kcal/mol (*SI Appendix*, Fig S3*C*), showing minimal changes from a complete PMF to a two-state model. We then utilized separate unbound and bound simulations with the absorbing boundary starting at 5 Å COM and obtained $\Delta G^{\circ }$ = −5.43 [−5.62, −5.24] kcal/mol (*SI Appendix*, Fig S3*D*). In addition, retaining the absorbing boundary starting at 5 Å and reducing the bound state to 0-2 Å COM produces a $\Delta G^{\circ }$ = −5.33 [−5.53, −5.14] (*SI Appendix*, Fig S3*E*). It was only when we reduced the absorbing boundary to 3 Å and defined the bound state at 0 to 2 Å COM ($\Delta G^{{}^{\circ}}$ = −4.77 [−4.98, −4.52] kcal/mol (*SI Appendix*, Fig S3*F*) that we observed a substantial change in the calculated $\Delta G^{{}^{\circ}}$, which was consistent with our application of absorbing boundaries to the full PMF (Fig. 2*E*). The optimal application of cutoffs without a loss in accuracy of binding free energy was to define the bound state from 0 to 2 Å COM distance and the absorbing boundary ≥5 Å COM movement. These parameter settings for all bound and unbound states were utilized in the rest of this manuscript.

The observations from benzamidine trypsin validate several aspects of our theory on a real protein–ligand system and provide guidance for applying ModBind_dG_ in practice. First, population counting converges significantly faster than energy-based calculations (18, 20). Second, because populations of the bound state (i.e., free energies) are essentially unchanged >5 to 10 Å, we can assume that the binding and unbinding process is quasi-Markovian (exceptions would be systems involving conformational changes, occluded pockets, or other special circumstances). Therefore, a) simulations can be discretized into multiple states with no path dependence, only probabilities of state transitions, and b) forward and reverse pathways are symmetric regardless of starting position (34). This allows us to apply a two-state model to our ModBind_dG_ simulations, provided that the ligand is unlikely to rebind after moving from the bound state to an absorbing boundary. Third, using this two-state model, we can now run separate simulations of the ligand in the bound and unbound state, leading to a significant decrease in computational cost by stopping our bound state simulations between 5 to 10 Å and simulating the unbound state in a water box. Finally, our approach requires no prior knowledge of the reaction coordinate associated with ligand unbinding. This is unlike most geometric unbinding methods, where the unbinding pathway (i.e., a specific reaction coordinate) must be carefully chosen and the results can vary significantly depending on how closely the reaction coordinate represents the true pathways (35, 53).

Some assumptions accompany the ModBind_dG_ approach. (See *SI Appendix* for more detailed explanations.)

1. First, it is important to choose a reasonable coordinate system with well-defined bound and unbound states that describes the configurational change of interest, and the regions of the energy surface that meaningfully contribute to population statistics. This is a general requirement of population reweighting and related methods (21, 33). We follow the assumption by Ytreberg and Zuckerman that the undescribed collective variables of the system contribute a constant entropy to the states along a reaction coordinate (32). Population reweighting can use as many dimensions as needed to meet this assumption, but careful selection is suggested because more sampling is required to converge the bins in additional dimensions (21, 32, 33).
2. Bin sizes should be equal in phase space volume. In our case, we used Cartesian cubic coordinates of fixed size for reweighting. Additionally, the binning procedure constitutes a Riemann approximation of the integral, and bins should be small enough to follow the curvature of the energy surface but large enough for the ratios to be well-converged. Our method is relatively robust to changes in bin size changes and provides consistent predictions across approximately two orders of magnitude.
3. The frame capture interval must be small enough to accurately estimate the relative free energy difference between states after reweighting. Too small a frame capture rate will not affect the $\Delta G^{\circ }$, as the population ratio in Eq. **14** cancels this factor out but has practical limitations in terms of disk space and simulation time.
4. The number of trajectories does not affect the computed $\Delta G^{\circ }$ (i.e., the population ratio remains unchanged in Eq. **14**), but it does impact the statistical certainty of $\Delta G^{\circ }$ (Fig. 2 *C*, *D*, and *F* and *SI Appendix*, Fig. S3). This is also demonstrated in the PMF computed with varying numbers of trajectories (*SI Appendix*, Fig. S4).
5. The volume between absorbing boundaries must be chosen so that simulations proceed far enough along the reaction coordinate that the ligand has a high probability of transitioning states before being absorbed. The theory is absolute as $q\left(\vec{r}\right)\to 1$ or the volume between absorbing boundaries approaches zero. In practice, $q\left(\vec{r}\right)\approx 0.5$ is roughly equivalent to the statistical error of our calculations, significantly reducing computational cost with minimal impact on the absolute calculated $\Delta G^{\circ }$.
6. At higher temperatures proteins can undergo partial unfolding; thus, restraints were applied to the protein backbone distal from the binding site. We (19) and others (22) have used this protocol for off-rate predictions with good success, but it is possible that the restraints may introduce artifacts in ligand unbinding especially if the protein requires long range secondary structural rearrangement to facilitate unbinding. Care should be taken for targets without a solvent accessible binding pocket.

### ModBind_dG_ Applied to Pharmaceutical Targets.

**Fig. 3.**
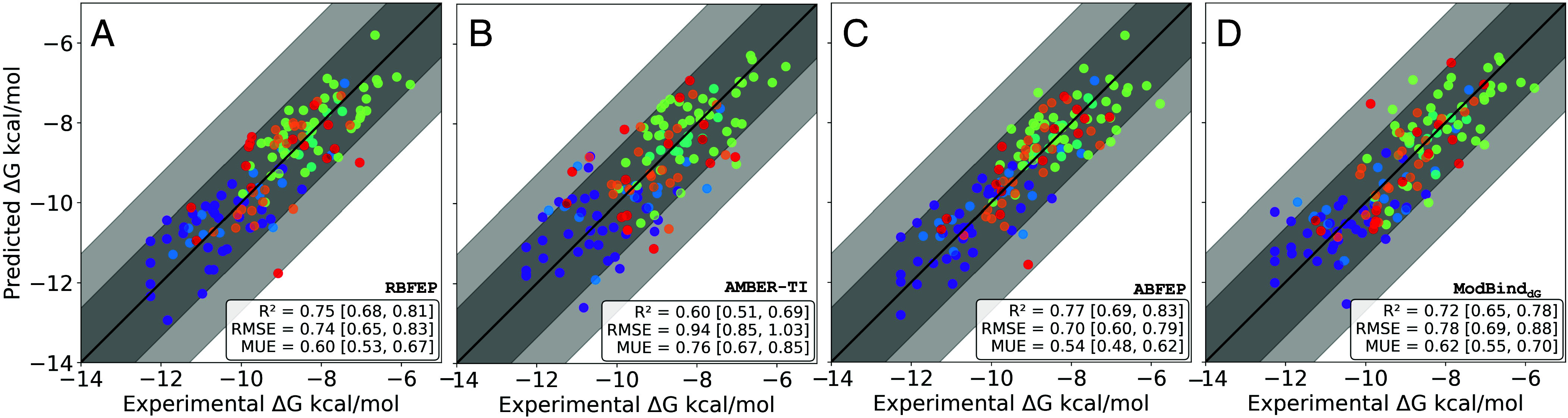
ModBind_dG_ is competitive with state of the art free energy methods. (*A*) RBFEP predictions versus experimental binding free energies from Wang et al. (2). (*B*) AMBER-TI predictions versus experimental binding free energies from Song et al. (4). (*C*) ABFEP predictions versus experimental binding free energies from Chen et al. (19). (*D*) ModBind_dG_ predictions. versus experimental binding free energies. *blue*: TYK2, *cyan*: thrombin, *lime*: MCL1, *orange*: JNK1, *red*: CDK2, *purple*: p38α kinase. Data have been normalized according to the mean and range (details are given in the *SI Appendix*). A 95% CI is given for all statistics displayed in brackets derived from bootstrapping the data.

Validation of ModBind_dG_ was carried out on a common free energy dataset composed of 8 targets and 199 ligands, initially used in relative binding free energy perturbation (RBFEP) (2) and used in a follow-up study for absolute binding free energy perturbation (ABFEP) (19). We did not include PTP1B and BACE in this correlation analysis as they involve loop movements and protonation state changes which make them challenging in models of ligand dissociation (19). ModBind_dG_ competes well against ABFEP (19) in terms of correlation (Fig. 3): When we normalize the data and plot TYK2, Thrombin, MCL1, JNK1, P38α, and CDK2, we obtain an *R*^2^ of 0.72 with an MUE of 0.62 against experiment, whereas ABFEP results have an *R*^2^ of 0.77 and an MUE of 0.54. This modestly higher correlation with ABFEP could be attributed to the difference in force field (the proprietary OPLS4 force field has historically outperformed GAFF with respect to small molecules) (54), or that this dataset was developed specifically for the FEP method (2, 19).

It is important to note that data in Fig. 3 and *SI Appendix*, Figs. S6 and S8 has been normalized for all compared methodologies, clearly showing that ModBind_dG_ is competitive with industry-standard approaches. What sets ModBind_dG_ apart is that we can accurately predict the absolute free energies of the ***entire*** dataset (including PTP1B and BACE) *with no normalization* (Table 1 and *SI Appendix*, Figs. S5, S7, and S9). ModBind_dG_ has an average error of 1.89 kcal/mol per target, whereas ABFEP has an average error of 3.99 kcal/mol per target (this difference is equivalent to a 24-fold error for ModBind_dG_ versus a 854-fold error for ABFEP in the predicted *K*_d_). In the case of ABFEP, a protein rearrangement factor is added to obtain agreement with experimental data; these correction factors need to be added on a case-by-case basis and do not fully explain the significant errors in predicted free energy values (up to 10 kcal-mol^−1^ ), and are often invalid against proteins that adopt different structures despite having the same binding site. These changes in protein conformations are difficult to calculate accurately due to energetic noise. Additionally, we note that the 95% CI for most of the normalized metrics for the six targets (Fig. 3 and *SI Appendix*, Figs. S4 and S6) are overlapping between ModBind_dG_ and ABFEP, suggesting that although normalized performance is generally slightly lower, the differences may not be large and varies target to target. However, the MUEs for the raw data are generally poorer for ABFEP (Table 1 and *SI Appendix*, Figs. S7 and S9).

**Table 1.** Comparison of mean unsigned error between ModBind_dG_ and ABFEP MUE data are reported as the absolute predicted value with no normalization for both methods

Target	ModBind absolute MUE kcal/mol	ABFEP absolute MUE kcal/mol
BACE1	2.19	2.92
CDK2	2.77	1.22
JNK1	0.76	3.50
MCL1	2.96	3.65
P38	0.84	5.27
PTP1B	2.17	9.97
Thrombin	1.84	1.82
Tyk2	1.58	3.54
Average 8 targets MUE kcal/mol	1.89	3.99
Average Fold Error (X)	**24.42**	**854.58**

All ABFEP were reported in Chen et al. (19).

Analyzing the results on a target-by-target basis reveals that high-temperature acceleration in ModBind_dG_ may reduce the range of predicted binding energies compared to experiment. Notably, targets run at higher temperatures (*SI Appendix*, Fig S10 and Table S1) predicted ranges lower than experiment especially CDK2 and P38, suggesting that we could increase our absolute correlation to experiment with a trade-off in simulation speed (*SI Appendix*, Figs. S5 and S7). We analyzed JNK1 absolute binding predictions across temperatures from 600 to 1,000 K and saw reasonable correlation from 600 to 800 K and absolute MUE; above 900 K we observed degradation in correlations and predicted ranges (*SI Appendix*, Fig S10). In addition, the absolute and normalized MUE values for ModBind_dG_ results are quite similar, indicating that ModBind_dG_ performs better at predicting absolute binding affinities in the same range as experiment compared to ABFEP.

We hypothesize that the improved absolute accuracy with ModBind_dG_ is due to our population sampling approach: Conformational populations converge much more rapidly than summation of energetic functions, as we have shown previously and in this manuscript (18, 20). In addition, our explicit treatment of the free energy contribution of the unbound state leads to improved absolute accuracy with ModBind_dG_. The unbound free energy of the drug-like ligands from the Wang et al. dataset has a narrow distribution, with an average value of −3.16 kcal/mol, a range of 0.55 kcal/mol, and variance of 0.09 kcal/mol (*SI Appendix*, Fig. S11). This behavior has little impact on the rank-ordering of compounds but is critical to the accuracy of absolute predictions due to its contribution to the ratio of bound to unbound states (Eq. **14**). Combined, these advantages make ModBind_dG_ particularly well-suited for applications in de novo drug design or virtual screening (VS) where compound activities for a given target are completely unknown.

**Fig. 4.**
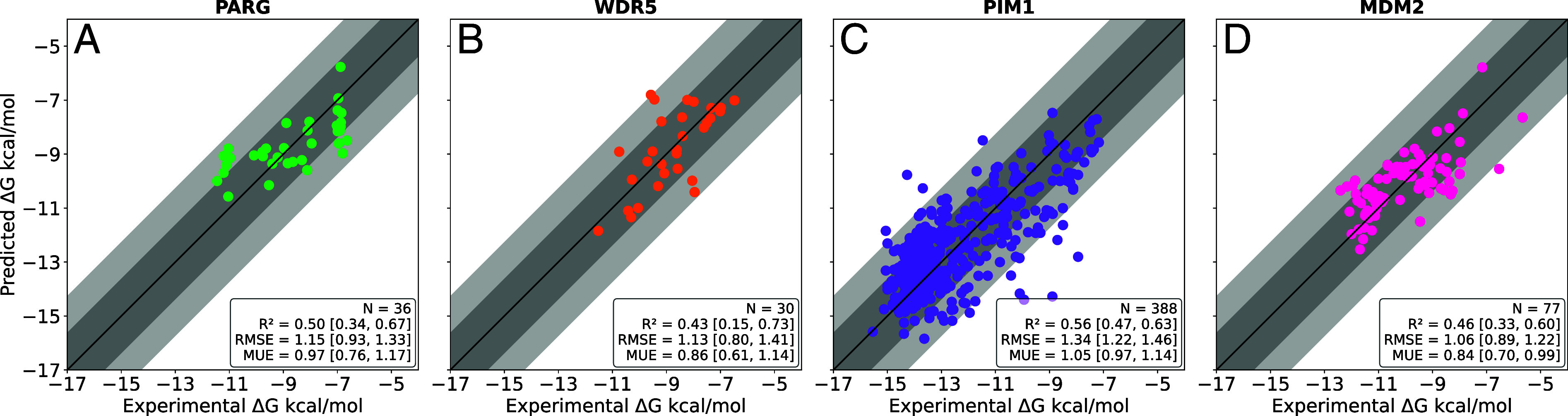
ModBind_dG_ is effective on internal drug discovery projects and broader datasets Correlation of ModBind_dG_ data versus experimental binding energies for (*A*) PARG, (*B*) WDR5, (*C*) PIM1, and (*D*) MDM2. Predicted free energies were calculated from Eq. **14**. Data were normalized according to the mean and range. N: number of compound predictions per target. 95% CI are shown in brackets as derived from bootstrapping.

We have developed a pipeline of drug discovery projects using computational chemistry and free energy methods, and ModBind (18) and ModBind_dG_ have been critical to the success of this pipeline. Two of our internal drug discovery programs are Poly (ADP-Ribose) Glycohydrolase (PARG) and WD Repeat protein 5 (WDR5), both of which are prominent oncology targets. ModBind_dG_ performs well for these real-world drug discovery targets against published experimental IC_50_ data with correlation of *R*^2^ = 0.50 for PARG and *R*^2^ = 0.43 for WDR5. (Fig. 4 *A* and *B*). Both datasets present similar correlation metrics to the maximum expected accuracy for computational free energy methods (55). Furthermore, we validated ModBind_dG_ on targets of general drug discovery interest including the Serine/threonine-protein kinase pim-1 (PIM1) and E3 ubiquitin-protein ligase Mdm2 (MDM2). These involved much larger datasets (N = 388 and N = 77 compounds) while maintaining good performance: *R*^2^ = 0.56 for PIM1 and *R*^2^ = 0.46 for MDM2 (Fig. 4 *C* and *D*).

### ModBind_dG_ Throughput, Virtual Screening, and Prospective Predictions.

Previously, we employed off-rate predictions using ModBind to rank compounds from a docking-based virtual screen, obtaining a 10× enrichment of novel chemical scaffold hit compounds as well as up to a 40% experimentally validated hit rate with the best scoring compounds (18). Likewise, ModBind_dG_ should increase the hit rate, with the key advantage of providing approximate absolute free energies for each compound, allowing for a reasonable prediction of potency levels.

To test ModBind_dG_ in a virtual screening campaign, we first docked the entire DUD-E (56, 57) library for p38 MAPK (36,379 compounds) using AutoDock to rank all molecules (*SI Appendix*, Fig. S12*A*). Within the top 100 hits, AutoDock ranking had captured 20 active compounds and 80 inactive decoys. While docking had enriched for active compounds, it failed to differentiate between active hits and decoys within the top 100 ligands ranked by docking score (*SI Appendix*, Fig. S12*B*). We then used ModBind_dG_ to rerank the same top 100 compounds resulting in a significant improvement in the enrichment factor (from 2.1 to 29, (*SI Appendix*, Fig. S12*C*), similar to what we achieved previously with ModBind. Of note, we were able to achieve these enrichment factors at an increased temperature all the way to 1,200 K, reduced the number of replicas to eight and estimated the unbound free energy based on the Einstein–Smoluchowski approximation. These changes reduced the total cumulative simulation time per ligand to ~0.5 ns, which translates into enabling the evaluation of ~2,000 compounds per day on a mid- to high-end GPU.

We then deployed ModBind_dG_ in a VS for a challenging undisclosed target with no previously known inhibitors and no known drug binding site. We chose a binding site based on site-finding analysis, where the protein geometry and local electrostatics could potentially enable small molecule binding. Next, we ran a docking-based VS of ten million compounds using Schrodinger Glide (39, 40) against the putative binding site. The top 10,000 compounds ranked by docking were then evaluated using ModBind_dG_ to predict binding energy and act as a secondary filter to inform purchases of compounds for testing. We identified ten novel compounds of varying chemotypes with inhibition <100 μM via surface plasmon resonance (SPR) assay. It took 2 mo including compound ordering and experimental validation from the initiation of the VS to identification of hits. This significant result stemmed from the combination of docking and ModBind_dG_ providing higher enrichment rates based on ModBind_dG_’s ability to accurately estimate absolute ligand binding free energies for de novo VS hits. We are unaware of any other computational methods that can provide the same level of accuracy, speed, and absolute prediction across varying chemotypes, making ModBind_dG_ ideal for identifying leads for previously undrugged proteins and binding sites.

### Comparison of ModBind_dG_ Throughput to Leading Free Energy Approaches.

In contrast to ModBind_dG_, most alchemical methods require between 640 to 1,240 ns per compound, (2, 4, 19, 58–60) making them significantly more computationally expensive (Table 2). ModBind_dG_ markedly outperforms other free energy techniques as it efficiently explores phase space via accelerated sampling; this avoids the lengthy phase space exploration characteristic of free energy methods. ModBind_dG_ converges rapidly as well; while we used 8 to 32 individual replicas for the ModBind_dG_ work presented in this manuscript, a converged result can often be found after only a few replicas. We analyzed representative results by calculating the convergence after adding each individual replica and found that between 0.5 ns and 10 ns of total simulation time was necessary to achieve a reasonably converged result (*SI Appendix*, Fig. S13). Additionally, the setup of our method is simple and does not rely on the complex construction of free energy maps between ligands as do relative free energy perturbation (61, 62) or thermodynamic integration. (4) ModBind_dG_ does not require any specific knowledge of the unbinding pathway or definition of a collective variable. This is a significant advantage over geometric binding methods because identifying an accurate, appropriate collective variable is nontrivial, and a poor selection can severely impact the accuracy of results.

**Table 2.** The estimated simulation time and number of compounds per day per GPU for alchemical free energy methods and ModBind_dG_ based on a hypothetical GPU that achieves a 1,000 ns/d

Method	ns/compound (ns)	compounds/d (#)	Reference ligand?	Absolute avg. error (fold)	Absolute avg. error (fold)
RBFEP	250–500	2–4	Yes	N/A	N/A
ABFEP	640–1,240	0.8–2	No	1,000×	3.99
ModBind_dG_	<1-4 ns	100–2,000	No	20×	1.89

ModBind_dG_’s use of a simplified two-state system enables further efficiencies because it avoids simulating regions of the free energy surface along the unbinding pathway that do not significantly contribute to the final free energy of binding. The use of the Einstein–Smoluchowski equation to estimate the free energy of ligand diffusion in bulk solvent further reduces the computational cost of ModBind_dG_. Perhaps the most encouraging aspect of ModBind_dG_ is its robust versatility: We have internally validated it against novel targets with no reported binders, tackled diverse chemical series and protein conformations, and can predict a broad range targets at different temperatures knowing that our predicted free energies will be consistent with experimental results.

Our method is also amenable to development of target and scaffold generalizable AI affinity models. AI model development requires large amounts of accurate and consistent data covering diverse chemical and target spaces, but such data fulfilling both quality and quantity are not currently present in the drug discovery field. With the speed, accuracy, and generalizability of ModBind_dG_, we are currently engaged in generation of very large synthetic affinity datasets to train predictive and generative AI models, enabling the large-scale AI training necessary to create truly generalizable AI models. Taken together, these theoretical and methodological advances to ModBind makes ModBind_dG_ an effective and practical choice for use in early to later stages in the drug discovery process.

## Conclusions

Here, we have presented key improvements to the population-based reweighting method and an update to our original ModBind algorithm, ModBind_dG_. ModBind_dG_ is an absolute binding free energy method based on rigorous statistical mechanical principles. We show that under certain conditions common to drug discovery settings, a discrete two-state system can be used to predict free energy differences of bound and unbound states, eliminating the need to simulate the extremely long timescales necessary to capture multiple binding and unbinding events for many systems relevant for drug discovery. Population-based reweighting of the two-state system connected by absorbing boundaries enables faster convergence and up to a 2,000× speedup without sacrificing accuracy. We validated ModBind_dG_ across 12 protein targets and more than 700 compounds showing best-in-class absolute accuracy (with no correction factor needed) compared to experiment. The utility of ModBind_dG_ has led to its implementation as a key tool in our drug discovery pipeline up to candidate nomination including for our WDR5 and PARG programs. In these and other cases, ModBind_dG_ achieved sufficient correlation with experimental data to enable efficient candidate discovery. Finally, we demonstrated that ModBind_dG_ can identify multiple novel hit compounds with drug-like properties when used on a challenging drug target, enabling the rapid start (<2 mo) of a drug discovery program from scratch, with sufficient speed to predict tens of thousands of compounds with reasonable computing resources. Since ModBind_dG_ is an absolute predictor exhibiting a balance of throughput and accuracy, we expect it to have widespread adoption in early hit finding and lead-optimization up to candidate nomination. Future developments of ModBind_dG_ include enhancements in speed, accuracy, and generalizability of the method as well as applications to synthetic dataset generation for AI training. In addition, we are applying ModBind_dG_ to efficiently optimize target specificity, as well as for prediction of small molecule solubility.

## Supplementary Material

Appendix 01 (PDF)

Dataset S01 (GZ)

## Data Availability

All the data and simulation details to run and validate our method is disclosed in the materials and methods, a previous related publication (18), or SI sections. We discuss one prospective drug discovery project where the data cannot be disclosed at this time for confidentiality reasons. We show numerous retrospective validation examples (12 protein targets and greater than 700 ligands) where we have disclosed all of the data, this makes up the bulk of the manuscript). Previously published data were used for this work (10.1021/acs.jcim.4c01805). Other data are included in the article and/or supporting information.
