# Supplementary material for: ModBinddG: A simulation-based absolute predictor of free energy of binding based on population reweighting
Source: Proc Natl Acad Sci U S A. 2026 Jun 15;123(25):e2513285123. doi: 10.1073/pnas.2513285123 (PMC13291613; doi:10.1073/pnas.2513285123)
Supplement: Supplementary file 1 — Appendix 01 (PDF) [file pnas.2513285123.sapp.pdf]

ModBind$_{dG}$ a Simulation-Based Absolute Predictor of Free Energy of Binding Based on Population Reweighting

William Sinko[1*], Blake Mertz[1], Yoh Terada[2], S. Roy Kimura[2]

1. Alivexis Inc. 1 Broadway, 14th Floor, Cambridge, MA 02142
2. Alivexis Inc. Daiichi Hibiya Building 7F, Shimbashi 1-18-21, Minato-ku, Tokyo 105-0004

* Email: sinko@alivexis.com

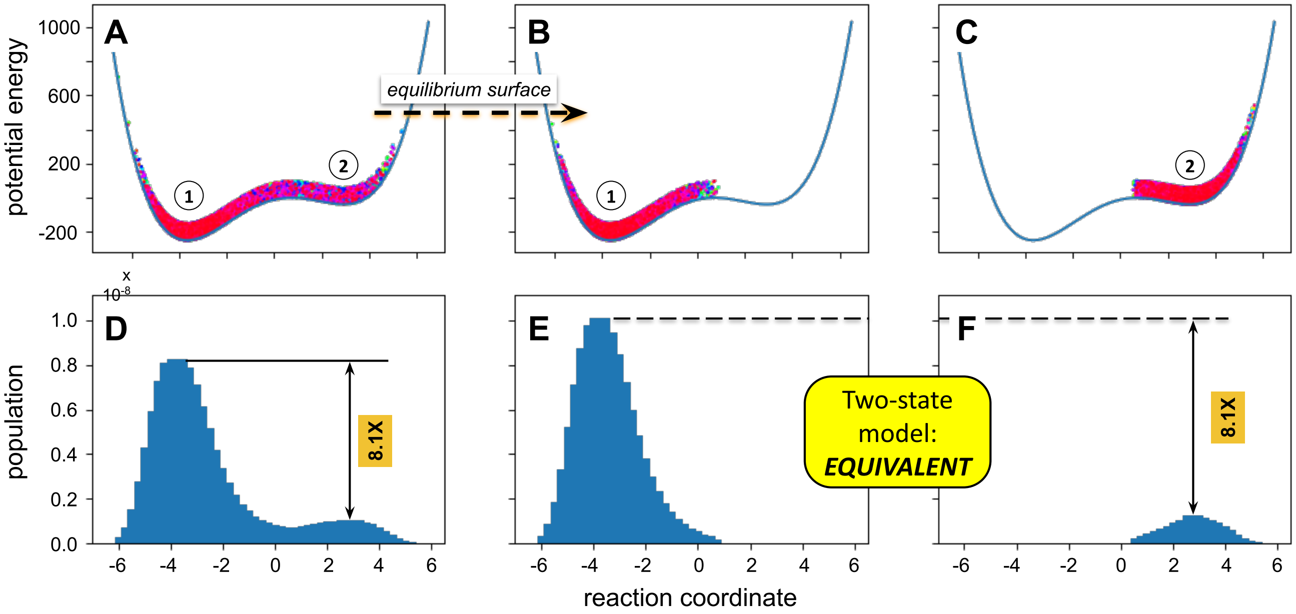

Figure S1. Two-well systems converge to the same distribution ratios as continuous systems when barrier crossings are properly accounted for. A) Plot of particle positions from an equilibrium molecular dynamics simulation of a particle along a 1-D coordinate with a two-well energy surface (states 1 and 2). B) Plot of particle positions from an equilibrium MD simulation of a particle with starting positions proximal to state 1. Simulations are restarted from the base of the state 1 well if they cross the energy barrier at the transition state (E = 0, reaction coordinate ≈ 1). C) Plot of particle positions from an equilibrium MD simulation of a particle with starting positions proximal to state 2. Simulations are restarted from the base of the state 2 well if they cross the energy barrier at the transition state (E = 0, reaction coordinate ≈ 1). D) Probability distribution of the MD simulation in A. E) Probability distribution of the MD simulation in B. F) Normalized probability distribution of the MD simulation in C. 10,000 particle starting points are simulated for 100,000 simulation steps each. Representative snapshot of 10,000 particle positions is shown in A, B, and C. The normalization procedure in panel F is to the ratio of escape events from (escapes well1/ escapes well2), this is multiplied by the raw populations state 2. Equivalently one could run the same number of simulations for panel E and F.

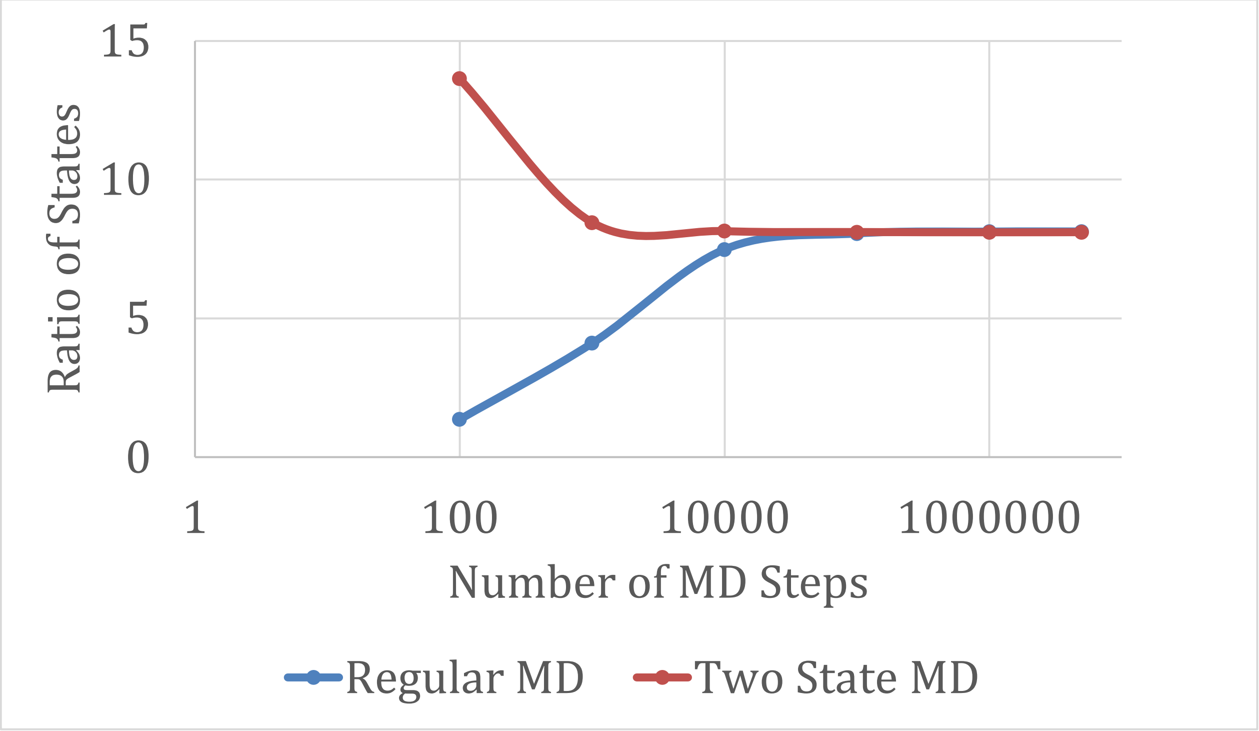

Figure S2. Two-state MD simulations converge more rapidly than continuous MD simulations on a 1-D energy surface. Ratio of state 1 to state 2 as a function of the number of steps in the MD simulation as described in **Fig. S1**. Both approaches are very close to convergence within 10,000 MD steps. However, the two-state MD approach converges more quickly (within 1000 steps) than the continuous simulation. For the two-state MD simulations, simulations are restarted if they escape their energy well

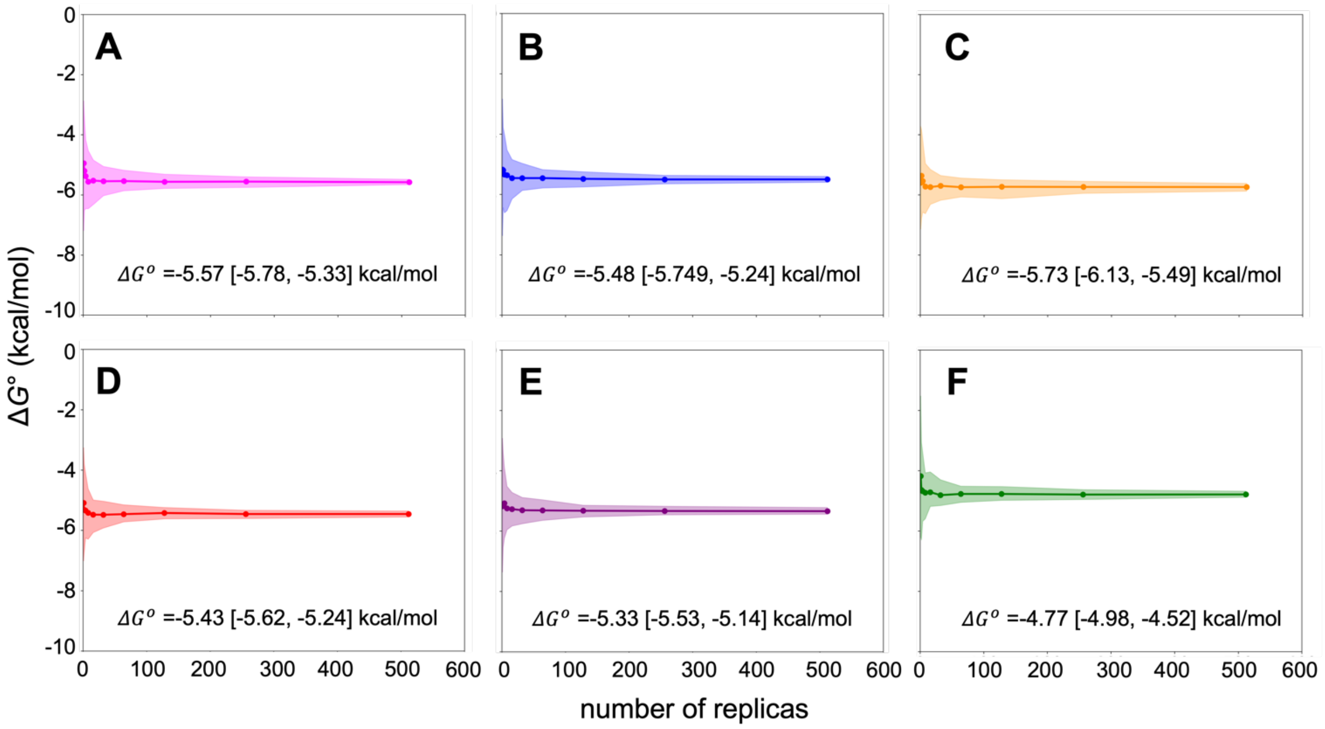

Figure S3. Free energy of scaled MD simulations of unbinding can be approximated with a dividing surface and bound state of varying length and separate unbound/bound simulations. Standard state free energy ΔG° (defined in Eq. 14 in the main text) as a function of the number of replicas.. **A)** Standard state free energy derived from applying a dividing surface from 20-30 Å (i.e., deletion of corresponding trajectory information). **B)** ΔG° derived from applying a dividing surface from 10-30 Å (deletion of corresponding trajectory information). **C)** ΔG° derived from applying a dividing surface 20 Å from the initial coordinates of the protein-ligand complex and a separate set of simulations of the ligand in bulk solvent. **D)** Separate bound and unbound simulations with the dividing surface applied at 5 Å from the initial protein-ligand complex for the bound state. **E)** Separate bound and unbound simulations with the dividing surface applied at 5 Å and the bound state boundary conditions defined from 0-2 Å COM movement. **F)** Separate bound and unbound simulations with the dividing surface applied at 3 Å and the bound state defined from 0-2 Å COM movement. A total of 512 replicas for both the bound and unbound state were simulated. For all plots the lightly shaded regions represent the 95% CI of the mean, and the dark solid line is the mean value based on bootstrapping with replacement.

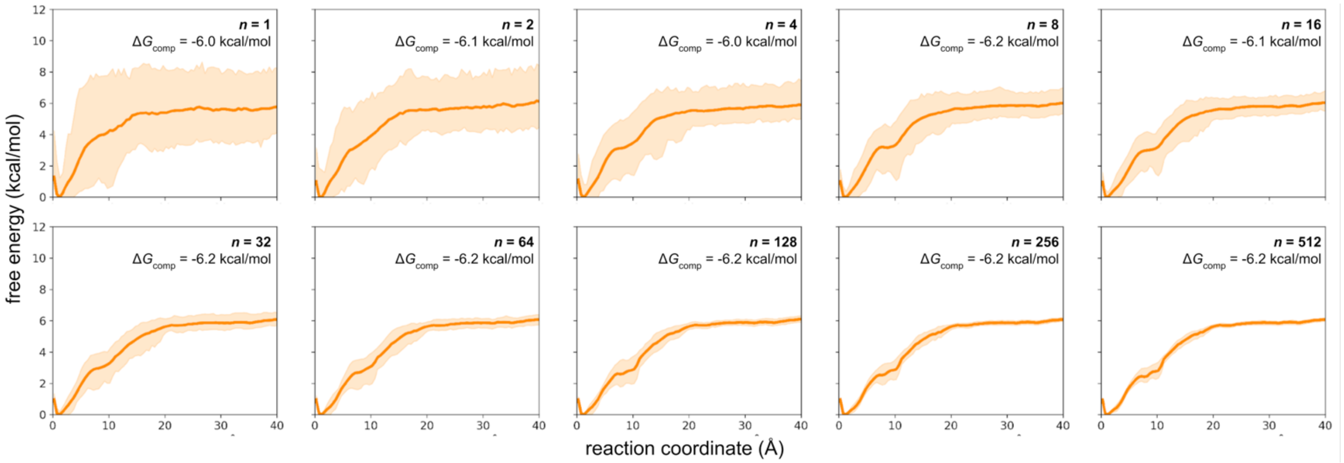

Figure S4: Potential of mean force (PMF) from high temperature scaled MD simulation reweighted via population-based reweighting as a function of replica number. The mean PMF (dark line) from 512 replicas total and pooled into n=1,2,4,8,16,32,64,128,256 and 512 replicas, the lightly shaded regions represent the 95% CI of the mean, means and CI are based on bootstrapping with replacement. ΔG_{comp} is computed as described in Eq. 11

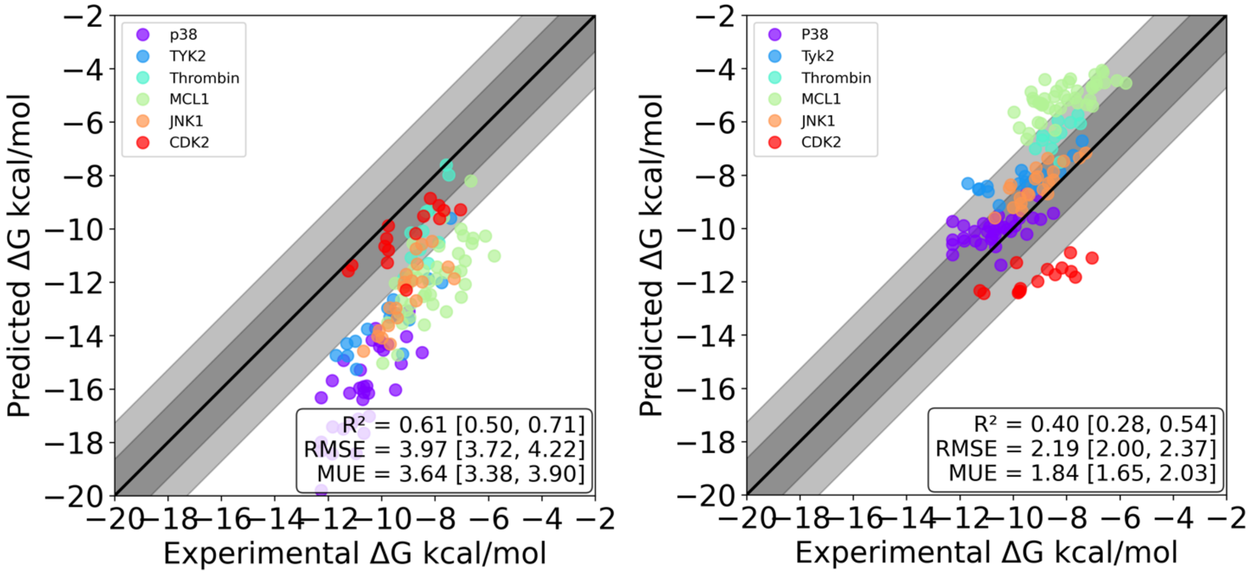

Figure S5. ModBind$_{dG}$ performs better than ABFEP with respect to ranges and error of predicted free energies. A) Non-normalized ABFEP predicted free energies versus experimental free energies from Chen et al. 2023.1 B) Corresponding non-normalized ModBind$_{dG}$ predicted free energies versus experimental free energies.

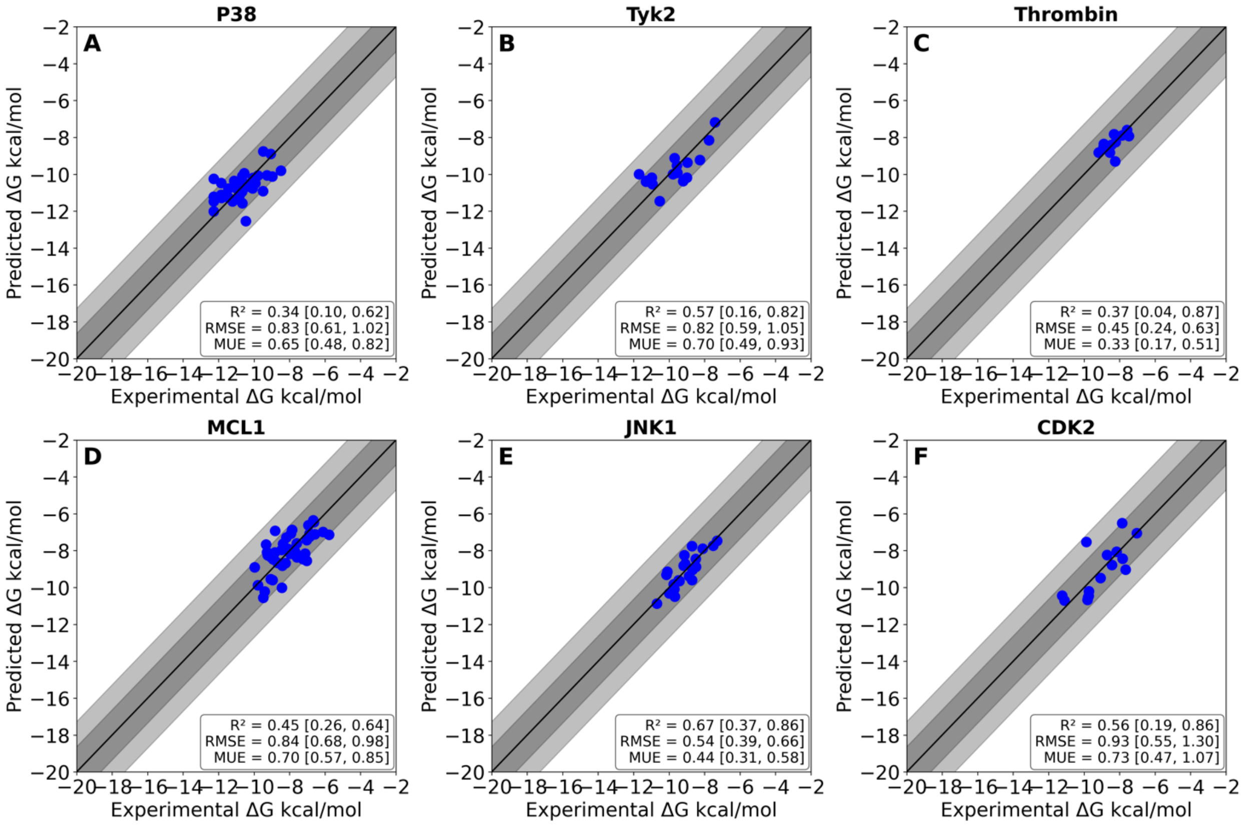

Figure S6 Normalized ModBind$_{dG}$ predictions per target. Normalized ModBind$_{dG}$ predicted free energies versus experimental free energies on targets on targets p38, TYK2, Thrombin, MCL1, JNK1, and CDK2. Correlation and error statistics are presented and 95% confidence intervals are given in brackets.

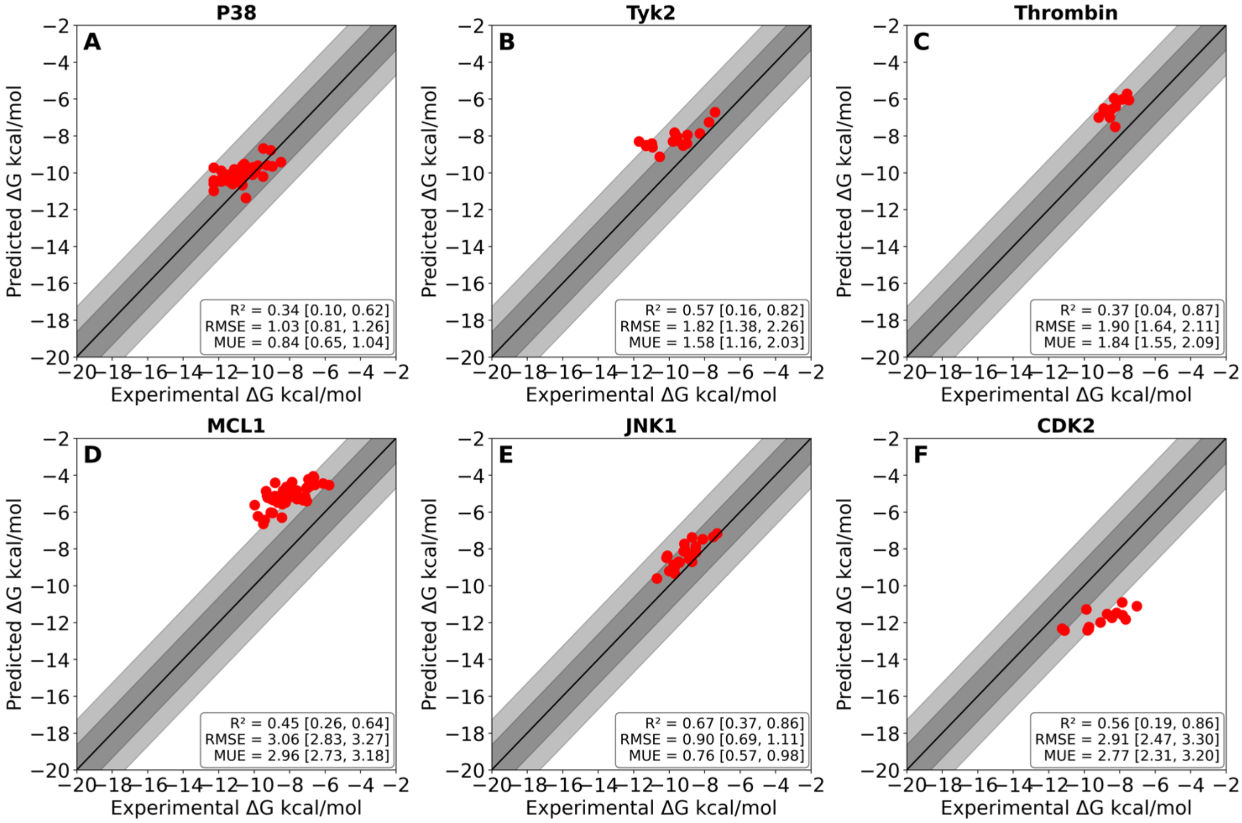

Figure S7 Raw ModBind$_{dG}$ predictions per target. Non-normalized ModBind$_{dG}$ predicted free energies versus experimental free energies on targets p38, TYK2, Thrombin, MCL1, JNK1, and CDK2. Correlation and error statistics are presented and 95% confidence intervals are given in brackets.

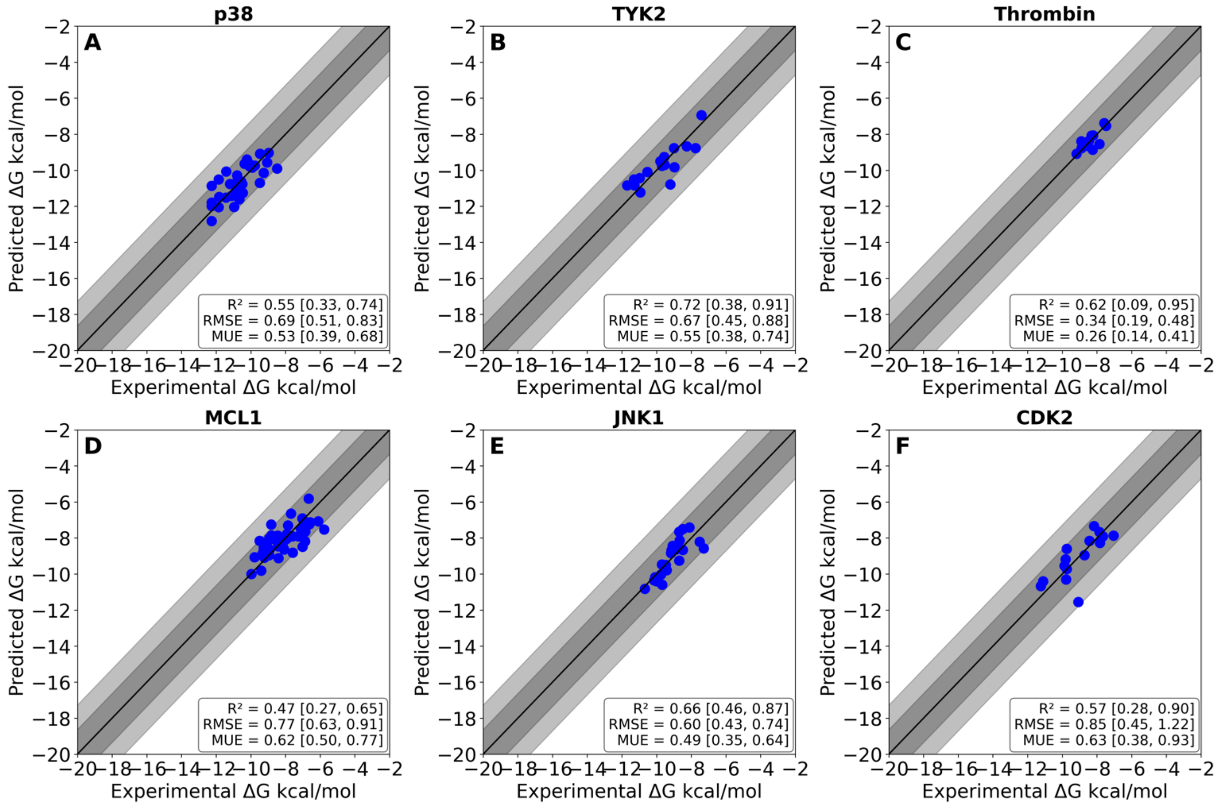

Figure S8 Normalized ABFEP predictions per target. Normalized ABFEP predicted free energies versus experimental free energies on targets p38, TYK2, Thrombin, MCL1, JNK1, and CDK2. Correlation and error statistics are presented and 95% confidence intervals are given in brackets.

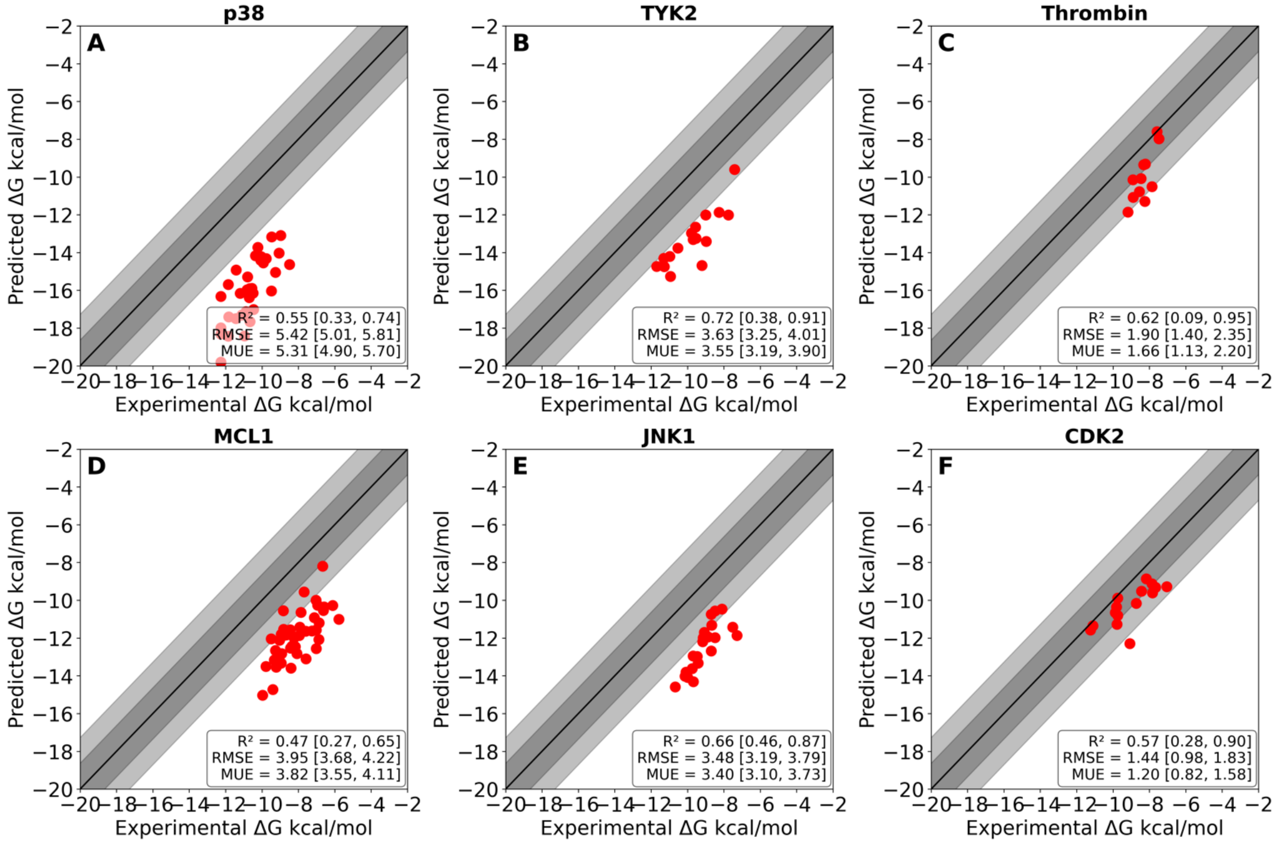

Figure S9 Raw ABFEP predictions per target. Non-normalized ABFEP predicted free energies versus experimental free energies on targets p38, TYK2, Thrombin, MCL1, JNK1, and CDK2. Correlation and error statistics are presented and 95% confidence intervals are given in brackets.

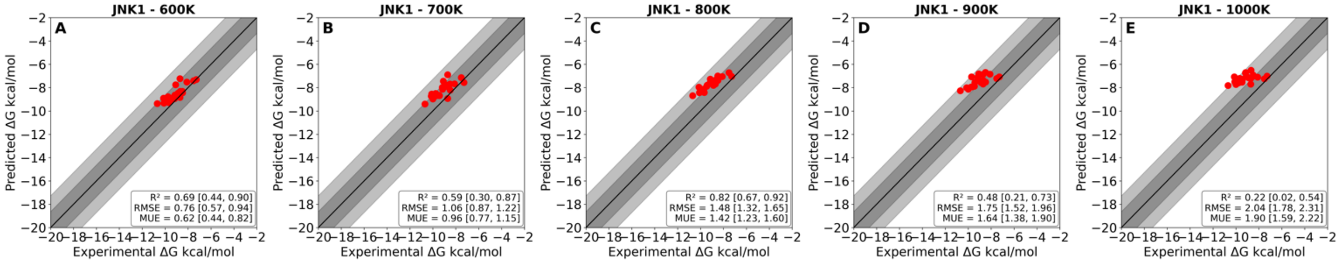

Figure S10 Raw ModBind$_{dG}$ predictions for JNK1 at varied temperatures from 600K-1000K. Non-normalized Raw ModBind$_{dG}$ predicted free energies versus experimental free energies for JNK1 from 600 K to 1000 K.

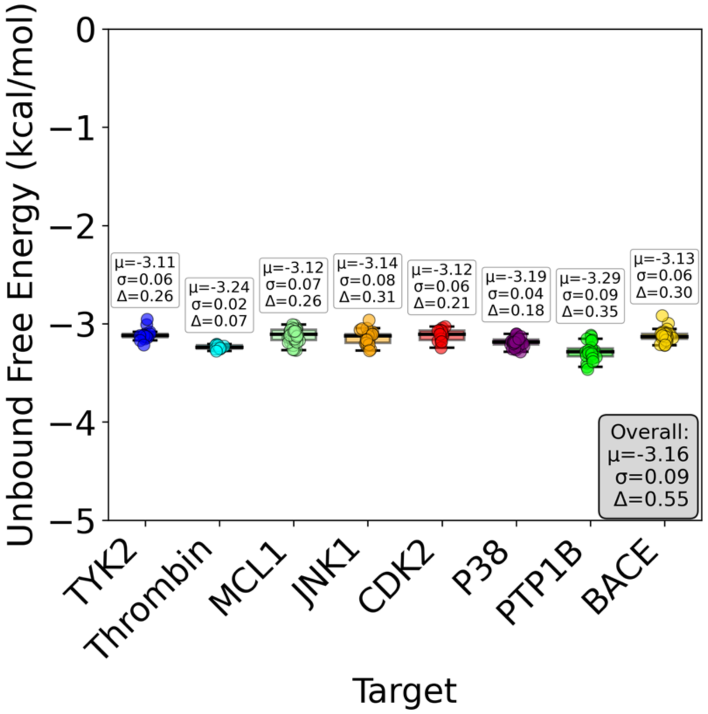

Figure S11 Unbound free energy predictions show little variation. p38a (purple), TYK2 (blue), Thrombin (cyan), MCL1 (mint), JNK1 (orange), CDK2 (red), BACE1 (gold), PTP1B (lime). µ (mean), σ (standard deviation), Δ (range) across all targets the range and standard deviation is low.

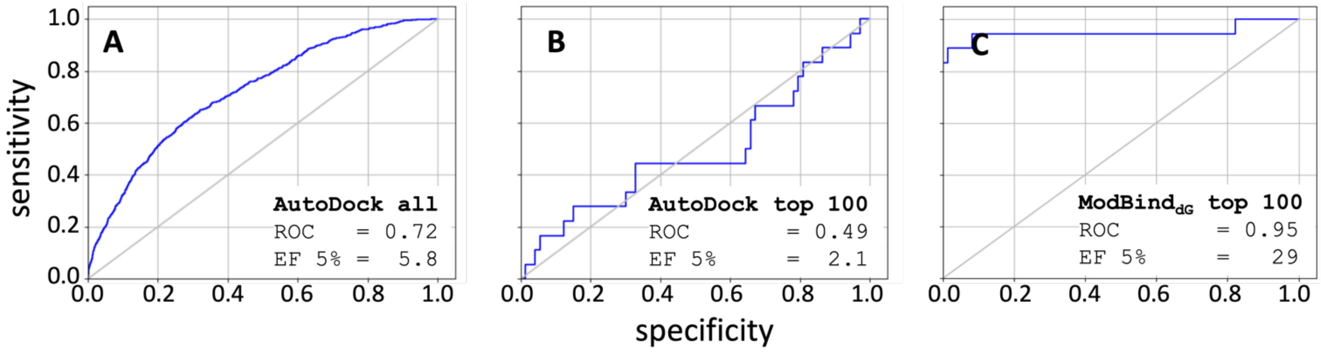

Figure S12. ModBind$_{dG}$ can further enrich the top docking hits from a VS campaign. A) The enrichment curve and ROC and EF 5% statistics for docking the complete 36,379 compound library for p38 MAPK kinase. B) The enrichment curve and ROC and EF 5% statistics when ranked by docking for the top 100 compounds by docking score. C) The enrichment curve and ROC and EF 5% statistics when ranked by ModBind$_{dG}$ for the top 100 compounds by docking score.

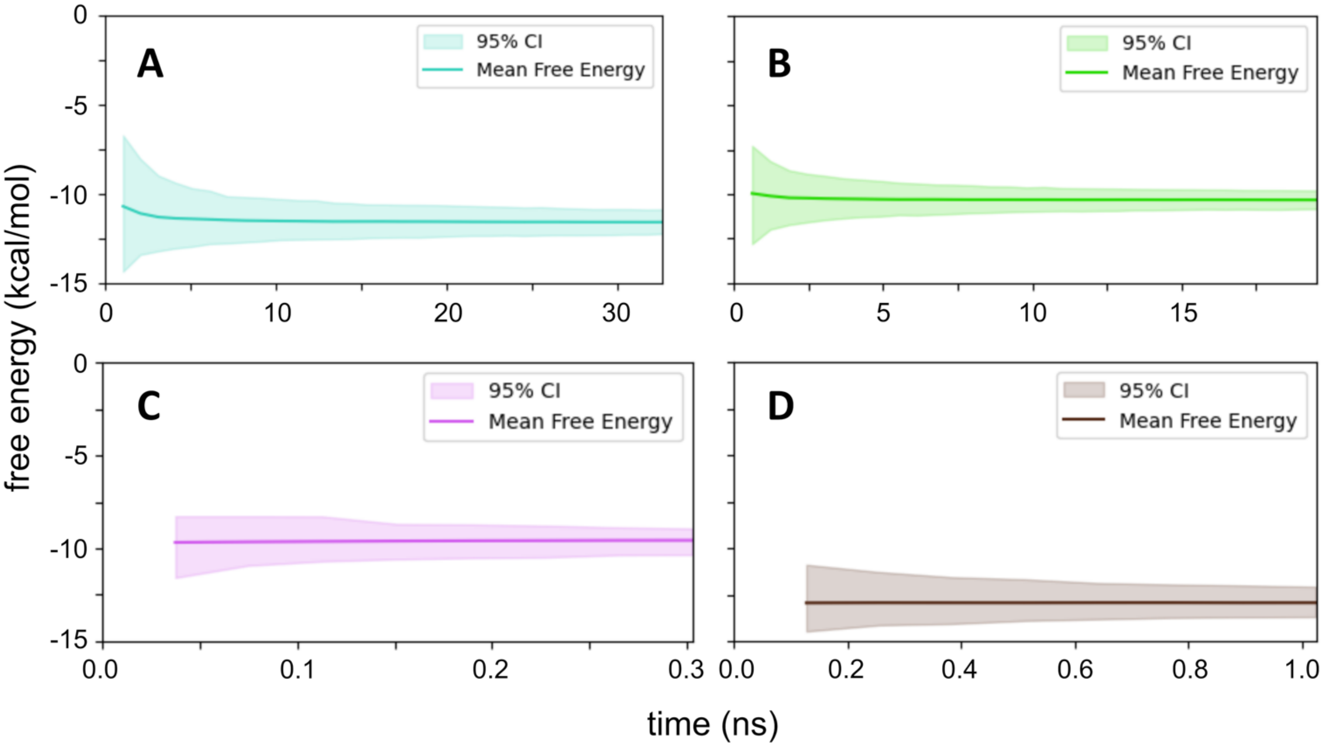

Figure S13 Convergence of bound state free energy in ModBind$_{dG}$ simulations representative examples. Convergence is calculated after each individual simulation is completed and ligand has escaped to the dividing surface. Dark line indicates mean and shaded area is 95% CI, and the x axis is total simulation time, bootstrapping varying numbers of replicas gives the measure of statistical uncertainty. A) JNK1 Ligand 17124-1 B) JNK1 Ligand 18636-1 C) P38 MAPK ligand CHEMBL559885 D) P38 MAPK ligand CHEMBL199679. The populations are normalized to the total number of trajectories to eliminate population drift as a function of summing over more trajectories by this equation $\mathrm{population_{normalized}} = \sum_i \mathrm{population}_i \cdot \frac{N_{\mathrm{ref}}}{N_i}$, prior to calculation of the bound free energy, since the unbound term in the ratio of **(eq 15)** is not included in this here.

METHODS

Generally, simulations were setup and run as previously described in our original ModBind paper.[2] Details are given below.

System setup. Unless otherwise noted, Schrodinger Glide[3] docking with maximum common substructure (MCS) alignment was used to align compounds to the corresponding crystal structure ligand for all initial poses in ModBind. Each compound in the dataset was inspected manually and confirmed to have a reasonable pose prior to predictions with ModBind. All protein structures were obtained from the Protein Data Bank (www.rcsb.org) unless otherwise specified. All 3D prepared protein structures used can be found in the *Supporting Information*. Protein structures were prepared using the Schrodinger protein preparation workflow[4] to model in missing residues, determine tautomeric and pK_a states, and perform a restrained minimization to optimize the structure before docking. If a known binding mode existed for a ligand to the protein target, 3D alignment and/or restrained docking was used. For targets without a known related ligand binding pose, docking was performed with ligands restricted to experimentally validated binding sites. Glide[3,5] or Autodock Vina[6] were used to generate all ligand-receptor poses unless otherwise noted. Ligand force field parameters were generated following the protocol implemented in OpenMM,[7,8] which uses the openmmforcefields package[9] to develop parameters consistent with the general Amber force field 2.1.[10] The protein force-field employed was ff14SB[11] and the water model utilized was TIP3P[12] for all simulations.

For PARG the compounds were selected from a published patent and aligned to the 5LHB crystal structure using MCS docking as input poses into ModBind$_{dG}$.[13,14] For WDR5 the 3D poses of the ligands were pulled from an alignment to the PDB structure 6DAR from BindingNet[15] with the original data originating from a study by Fesik and coworkers.[16] PIM1 structures obtained from BindingNet[15] and aligned via MCS docking prior to simulation as originally described by Do et al.[17] MDM2 structures were obtained from BindingNet[15] and used in the simulations as originally described by Berghausen et al[18]. For these four targets we used the Einstein-Smoluchowski approximation for the ligand free energy in the unbound state. For all targets binding modes and ligand states were visually inspected and modified in accordance with best practices for the setup of free energy methods.[19]

Temperature determination Temperatures in this study ranged from 550-1200 K and were maintained with the Langevin thermostat.[20] Individual simulation times depended on the rate of ligand unbinding, typically 1-5 ns or less. Temperatures and simulation times were optimized by running a small set of simulations on a known inhibitor(s), typically 1-2 compounds, and calculating the corresponding bound free energy varying the temperature by 50 Kelvin (an example is given in **Fig S11**). A temperature was

then selected based on spread of the predicted free energies and reasonable simulation time. Temperatures for all systems studied here are provided in **Table S1**.

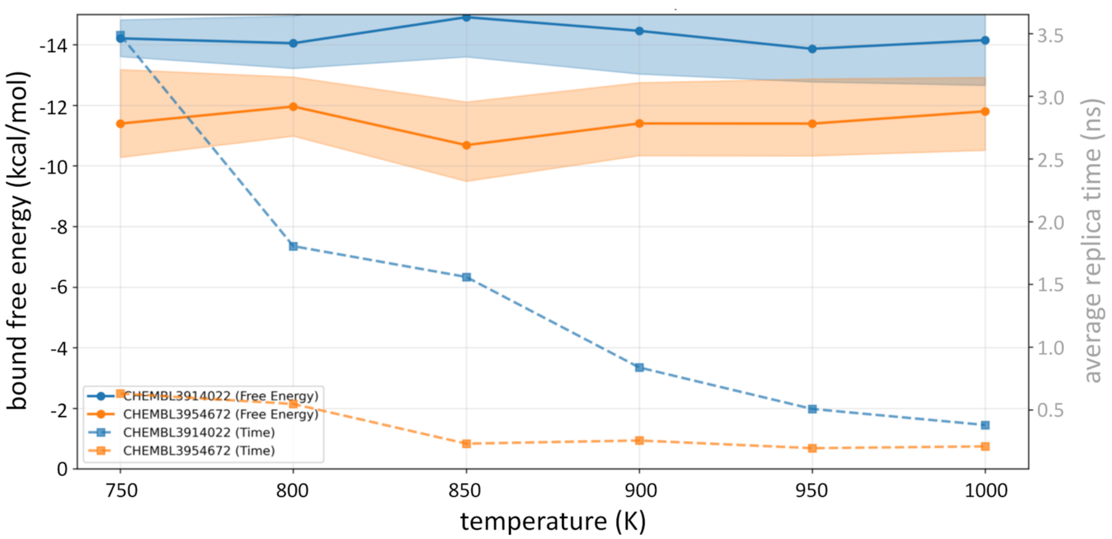

Figure S14 Free energy and simulation time of the bound state based on varied temperature for PIM1. Reasonable resolution of a weekly bound inhibitor CHEMBL3954672, and a tightly bound inhibitor CHEMBL3914022 are seen at temperatures 750-900K.

Table S1 Temperatures for all protein ligand test systems used in this manuscript.

**Target System**	***T* / K**
Trypsin	650
P38a (Wang et al.)	750
P38 (Virtual screen enrichment)	1200
TYK2	600
Thrombin	550
MCL1	600
JNK1	650
CDK2	750
PTP1B	650
BACE1	650
PARG	800
WDR5	800
PIM1	850
MDM2	850

Simulation parameters. Simulations were conducted in the *NVT* ensemble with a 2 fs timestep using OpenMM after minimization and equilibration using OpenMM[7]. The NVT ensemble must be used to retain standard density at the high temperatures of these simulations. Backbone atoms of the protein were restrained (default value of σ = 3.0 Å but may need to be adjusted depending on the system) except for residues within 6 Å of the protein binding site. The use of restraints is applied to prevent protein unfolding prior to unbinding, which likely would result in poorer prediction accuracy. We and others[21] have used restraints in unbinding simulations without seeing deleterious effects on either the prediction accuracy or the ability of a ligand to unbind across many targets. While we have not encountered issues with the restraints, there may be cases where restraining the protein could prevent the ligand from fully unbinding if the pathway is blocked by restrained protein residues. A simple visual inspection of the binding site will allow a practitioner to determine if it is feasible for a ligand to escape to solvent without major protein conformational change. Between 8 and 32 replicate trajectories were run to generate sufficient statistics for reliable calculation of the free energy of binding. Unless otherwise specified, we used a center of mass (COM) based metric in Cartesian dimensions, defined as the distance between the initial and current positions of the COM of the ligand in each respective simulation. The COM metric has proven more robust than RMSD to large changes in compound size and is also more amenable to binning the simulation movement along a reaction coordinate. Additionally the 3 Cartesian dimensions of COM can easily be binned into equal volumes using cubes.

Analysis. Pytraj,[22] a Python package binding to the cpptraj program,[23] was used for post-processing simulations. Trajectory frames were aligned to the first frame (protein-to-protein) for each respective trajectory. The center of mass (COM) position, XYZ was then calculated for each respective ligand. Reweighting is carried out by using Eq. (4) with room temperature (T = 300 K).

Normalization procedure. To enable quantitative comparison between computational predictions and experimental measurements, a standardized normalization procedure was applied to all datasets. This approach addresses the inherent scale or errors between computational methods (such as ModBind, ModBind^{dG}, relative FEP, and AMBER TI and ABFEP). The normalization protocol consists of two sequential steps: (1) mean normalization, whereby the average values of computational and experimental datasets are set to be equal, and (2) range normalization, ensuring equivalent data spreads across methods. Mean normalization is required to relate relative binding affinity calculations, including free energy perturbation (FEP) and AMBER TI, to free energy of binding or the Ki. Importantly, this normalization preserves the relative ranking of ligands within each computational method, maintaining the integrity of method-specific binding predictions. To ensure unbiased comparative analysis, identical normalization procedures were applied to all approaches examined in this study, including ModBind, FEP, and AMBER TI. Correlation coefficients remain unchanged under this transformation on a per target basis, and MUE and RMSE may be compared as all computational approaches received the same normalization procedure. We did not apply this normalization procedure to Table 1 or Figure S3, S5 or S7 so that the non-normalized MUE could be compared between ABFEP and ModBind^{dG}.

Calculation of statistics. R^2 is calculated as the coefficient of determination from linear regression. RMSE is the mean squared error $\mathrm{RMSE} = \sqrt{\frac{1}{n}\sum_{i=1}^{n}(x_i - y_i)^2}$, and MUE is the mean unsigned error $\mathrm{MUE} = \frac{1}{n}\sum_{i=1}^{n}|x_i - y_i|$. The 95% confidence intervals for R^2, RMSE, and MUE, $\Delta G°$, ΔG_{comp}, PMFs and statistics are calculated using the bootstrap resampling method, following best practices established in computational molecular modeling literature[24,25]. Briefly the data is bootstrapped 1000 times with resampling, a 95% confidence interval is determined using the percentile method, taking the 2.5th and 97.5th percentiles of the 1000 bootstrap statistics.

PMF calculations of benzamidine trypsin. For the benzamidine trypsin test case using ModBind$_{dG}$, continuous potential of mean force (PMF) surfaces were generated by extending the MD simulations until the ligand had moved 50 Å in from the starting position. We used the PDB ID 3PTP structure as the starting structure with the ligand bound. We increased padding of the water box from 10 Å to 25 Å to ensure that the ligand was not interacting with periodic images of the protein upon dissociation. 512 replicate simulations were run, using the same starting position for all simulations, but different randomized velocities. Reweighting of sampled populations was performed by creating a 3-D histogram of the populations in Cartesian space based on the COM distance of the ligand from its starting bound position and then applying **eq. 4**. ΔG^o was calculated as given in **Eq. 14**. Statistics were calculated via bootstrapping varying numbers of replicas for all convergence plots and a 95% CI was reported along with the mean. The bound state was defined as all populations within 5 Å COM distance from the starting position in the protein-ligand complex except as specified in **Fig. S3E** and **S3F,** which used a 2 Å COM distance and **Fig. 2E**) where the bound state and dividing surface were varied to test the impact on the calculated $\Delta G°$. The unbound state was defined as all populations within 5 Å of the position of the ligand after it first crossed 30 Å distance, or a separate unbound simulation with only the ligand in a box of water as specified in **(Figs. S3C. S3D, S3E,** and **S3F).**

Two-state ModBind$_{dG}$ Calculations. For the two-state ModBind$_{dG}$ simulations we ran a short unbinding simulation as described in Sinko *et al*.[2] and a companion simulation with the ligand alone in aqueous solution. The definition of the bound and unbound state were derived from our simulations of the benzamidine-trypsin system. The bound state was defined as 0-2 Å COM with the dividing surface at 5 Å or greater, both in Euclidian distances. After passing this point, simulations were stopped, and this was counted as an escape event. The unbound state was considered to be a 0-5 Å wide bin of COM movement in Euclidian distance in water without the protein present.

High temperatures for the ligand unbinding simulations were determined as described above, and ligand-only simulations were run at T = 300 K. Ligand unbinding from the protein complex was characterized as the change in distance of the ligand COM as a function of starting position, with the lowest energy state always occurring ≤2 Å COM. Binned populations within this COM threshold were used to calculate the bound term of Eq. 14. For the ligand in aqueous solvent, simulations were run for 10 ns. An escape event was quantified as the simulation progressing 5 Å movement in COM space from its origin; each time the ligand crossed the 5 Å threshold, the origin of the ligand was reset to its current position; this essentially mimics running multiple trajectories. This reset was carried out to ensure equivalent comparisons in COM movement between the ligand unbinding and aqueous simulations (i.e., at larger COM values the ligand can sample more volumetric space if allowed to freely diffuse). If the ligand in solution had more or less escape events to the dividing surface than the bound state simulations, we normalized the weights of the populations by the ratio of bound state escape events over unbound state escape events. Another alternative to explicit simulation of the ligand in aqueous solution is to use the Einstein-Smoluchowski equation to calculate the diffusion rate (see discussion below). The explicit approach was used over the Einstein-Smoluchowski approximation in this study unless otherwise specified.

***Binning, population-based reweighting procedure, and* ΔG° calculation.** A 3-dimensional (XYZ) histogram of Cartesian COM coordinates was constructed by binning the Cartesian values of COM distance from the starting position, using uniform cubes of 4 Å sides, and a constant volume of 64 Å^3. Bin reweighting is carried out by using Eq. (4) with room temperature (T = 300 K). Each frame within a bin was then assigned an equal share of that bin's reweighted population as its statistical weight. Frames falling within the bound and unbound state definitions were summed by their assigned weights as shown in Eq. (14), giving the total reweighted population of each state. ΔG° was calculated from the ratio of these populations using Eq. (14). Our method shows

robustness across bin volumes from 3-125 Å^3 *(see SI Note on bin size in reweighting)*. The state definitions were uniform for all systems as described above.

Rapid theoretical estimate of unbound free energy. In our two-state model, the simulation of the ligand unbound state is the same as the simulation of diffusion and counting of sampled populations related to MD frame capture rate and binning size. So to further accelerate calculations, it is reasonable to estimate the free energy of the ligand in solution using the Stokes–Einstein–Sutherland equation to estimate the diffusion coefficient. This approximation however treats the ligand as a hard sphere and disregards conformational and rotational degrees of freedom. For drug-like molecules this is a reasonable approximation, but in the case of large changes in molecular weight or very flexible ligands, it is suggested to explicitly model the unbound state via MD simulation. The diffusion constant *D* can be estimated by

$$D = \frac{k_B T}{6\pi\eta r} \quad \text{(SI-1)}$$

where k_B is the Boltzmann constant, *T* is the temperature in Kelvin, η is the viscosity of the medium (usually water for biological systems), and *r* is the radius of the molecule. Although precise determination of molecular radius is impractical for flexible non-spherical molecules, a reasonable approximation for drug-like molecules is D=0.5 x 10^{-9} m, consistent with published experimental and predicted values for drug-like molecules[26,27]. The mean squared displacement in 3D is given by the Einstein-Smoluchowski equation:

$$\langle r^2 \rangle = 6Dt \quad \text{(SI-2)}$$

where *t* is the time. We can rearrange this equation to obtain *t* for any distance of *r*.:

$$t = \frac{\langle r^2 \rangle}{6D} \quad \text{(SI-3)}$$

If we use a radius of 5 Å for the unbound state consistent with our all of our simulations in this manuscript, a drug-like molecule is likely to diffuse the distance in t = 0.0833ns. If we use 32 replicas of simulation until escape and record frames every 0.01 ns in our bound simulations we apply the same parameters (frame capture interval, number replicas) to our unbound term. This gives us a free energy of the unbound term.

$$G_{\text{unbound}} = -RT \ln\left(\frac{32\ replicas \times t}{0.01\ \text{ns frame capture rate}}\right) = -3.31\ \text{kcal/mol} \quad \text{(SI-4)}$$

The terms inside the parenthesis provide the raw population count with identical simulation parameters. This value is in excellent agreement with the free energies in solution that we obtained from our simulations. Simulations of ligands across our datasets do not show major significant variation in the free energy value for smaller ligands (i.e., satisfying Lipinski's rule of 5). Simulation or updated estimates based on the radius of a ligand may be necessary to apply this method to large molecules or biologics. Our initial tests on 199 diverse small molecules associated with the wang et al. dataset have a range of -2.96 to -3.46 kcal-mol^{-1} for 32 replicas with a mean of -3.16 kcal-mol^{-1} (Fig S8). Thus, applying an Einstein-Smoluchowski diffusion estimation of the unbound population or unbound free energy, should be a good estimation of the unbound state free energy rather than running simulations.

A note on timesteps and frame rate. We utilized Region-specific frame capture intervals were determined from autocorrelation analysis of 512 independent ligand unbinding trajectories for the benzamidine trypsin system at 650K. Generally we followed the guidelines of Grossfield et al.[24] It is noted that for a two state model the bound and unbound simulations are separate and will have separate effective independent samples N_{ind} which are calculated as follows:

$$N_{\text{ind}} = \frac{N}{2\tau_{\text{int}}} \quad \text{(SI-5)}$$

where *N* is the number of frames and τ_{int} is the integrated autocorrelation time calculated as:

$$\tau_{int} = 1 + 2\sum_{j=1}^{N_{max}} C_j \quad \text{(SI-6)}$$

where N_{max} is the maximum lag for summation and C_j the autocorrelation at lag index *j* is:

$$C_j = \frac{(x_k-\bar{x})(x_{k+j}-\bar{x})}{s(x)^2} \tag{SI-7}$$

where k is the observable value at a time step and the denominator is the sample variance.

For the bound region (COM < 20 Å), the autocorrelation time is longer of τ_{int} = 186.9±13.3ps than for the unbound region (COM > 30 Å from initial position) τ_{int} = 8.2±1.1 ps. Because we need to sample the populations of both states independently in the two state model of $\text{ModBind}_{\text{dG}}$ to converge the histogram it is necessary to separate the data as such. N_{ind}=7.9±0.5 (2432 frames in total) and 74.8±18.3 (229 frames total) for the bound and unbound portions of the simulation respectively. So, in general even at 1ps frame capture we are only ~3X over the N_{ind} sampling interval. Thus we gathered a reasonable maximum sampling rate of 1 ps for the unbound state and 10 ps for the bound state (saved less frequently for practical considerations). Since there is a difference in the state definitions the unbound state populations were normalized by multiplying the raw population count of the unbound state by the ratio of $\frac{bound\ frame\ capture\ interval}{unbound\ frame\ capture\ interval}$ so that the time spent in any configuration in the simulation is equivalent regardless of frame capture interval, in other words we have normalized t_i from equation 3. It is notable that for enhanced sampling simulations such as this it is recommended to run multiple replicas to get statistical certainty.[24] Furthermore sampling at faster frame capture interval will not cause a difference in the final free energy because the absolute population differences will cancel in the ratio between bound and unbound states of equation 15. See derivation below.

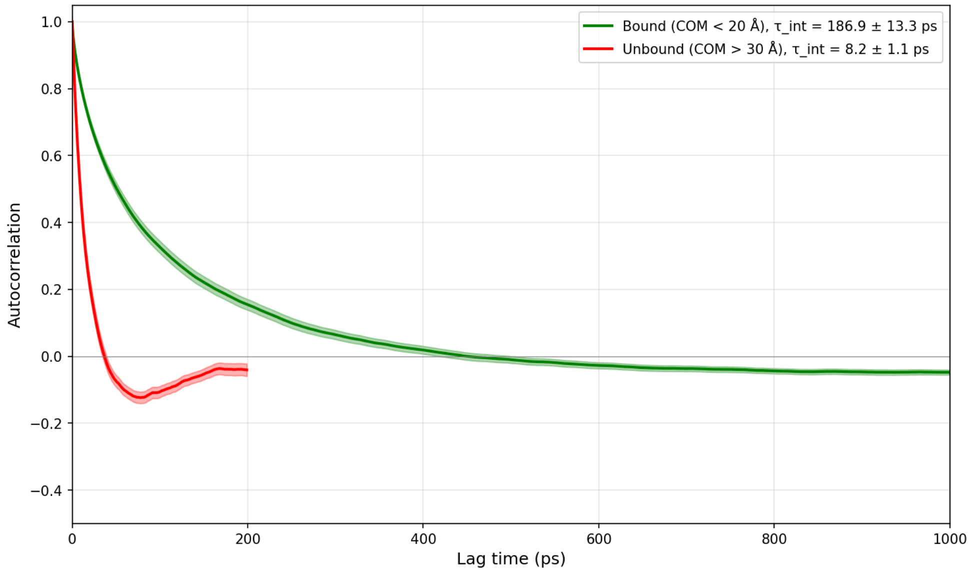

Figure S15 Autocorrelation function plotted against lag time for benzamidine trypsin system as calculated by equation SI-7.

Note on timestep and number of trajectories. The number of trajectories and the timestep do not affect the final computed free energy beyond statistical uncertainty. The driving reason is that both of these cancel in the ratio of populations used to compute any free energy (Eqs. 5 and 15) If we manipulate Eq 15 to explicitly show $t^*_{i,k}$, which is the time spent in state I in trajectory k, as well as $\sum_{k=1}^{N_{\text{traj}}}$ which is the sum across the number of trajectories N_{traj} as sampled on the modified potential within in a sum of configurations across a sum of trajectories.

$$\Delta G^\circ = RT\ln\left(\frac{\sum_{i\in\text{unbound}}\sum_{k=1}^{N_{\text{traj}}}\left(\frac{t^*_{i,k}}{\Delta t}\right)^{1/\lambda}}{\sum_{j\in\text{bound}}\sum_{k=1}^{N_{\text{traj}}}\left(\frac{t^*_{j,k}}{\Delta t}\right)^{1/\lambda}}\right) - RT\ln\left(\frac{V_u}{V^\circ}\right) \tag{SI-9}$$

$\frac{1}{\Delta t}$ cancels algebraically and can be removed from the summation indicating the size of the frame capture rate does not affect the ratio of populations because a population is time spent in a given configuration divided by the frame capture rate. Equation SI-9 is equivalent to Eq. 14 just using time as proxy for population which is $\frac{t_{\vec{r}_i}}{\Delta t}$ from equation 3.

$$\Delta G^\circ = RT\ln\left(\frac{\left(\frac{1}{\Delta t}\right)^{1/\lambda}\sum_{i\in\text{unbound}}\sum_{k=1}^{N_{\text{traj}}}\left(t^*_{i,k}\right)^{1/\lambda}}{\left(\frac{1}{\Delta t}\right)^{1/\lambda}\sum_{j\in\text{bound}}\sum_{k=1}^{N_{\text{traj}}}\left(t^*_{j,k}\right)^{1/\lambda}}\right) - RT\ln\left(\frac{V_u}{V^\circ}\right) = RT\ln\left(\frac{\sum_{i\in\text{unbound}}\sum_{k=1}^{N_{\text{traj}}}\left(t^*_{i,k}\right)^{1/\lambda}}{\sum_{j\in\text{bound}}\sum_{k=1}^{N_{\text{traj}}}\left(t^*_{j,k}\right)^{1/\lambda}}\right) - RT\ln\left(\frac{V_u}{V^\circ}\right) \tag{SI-10}$$

Given ergodic sampling for each trajectory, which is less likely in our case, or the law of large numbers (LLN) which we show here where the mean converges to the expected value. Then the number of trajectories N_{traj} can also be multiplied by $\frac{1}{N_{\text{traj}}}$ in the numerator and denominator for an expected value $E\left[\sum_{i\in\text{unbound}}\left(p^*(\vec{r_i})\right)^{1/\lambda}\right]$ and $E\left[\sum_{j\in\text{bound}}\left(p^*(\vec{r_j})\right)^{1/\lambda}\right]$ given that $N_{\text{traj}} \to \infty$. This effectively turns our numerator and denominator into a mean, which converges to the expected value as $N_{\text{traj}} \to \infty$. $\left(p^*(\overrightarrow{r_{k,i}})\right)$ is the modified populations of configuration i summed across k trajectories or N_{traj}.

$$\frac{\sum_{k=1}^{N_{\text{traj}}}\sum_{i\in\text{unbound}}\left(p^*(\overrightarrow{r_{k,i}})\right)^{1/\lambda}}{\sum_{k=1}^{N_{\text{traj}}}\sum_{j\in\text{bound}}\left(p^*(\overrightarrow{r_{k,j}})\right)^{1/\lambda}} = \frac{\frac{1}{N_{\text{traj}}}\sum_{k=1}^{N_{\text{traj}}}\sum_{i\in\text{unbound}}\left(p^*(\overrightarrow{r_{k,i}})\right)^{1/\lambda}}{\frac{1}{N_{\text{traj}}}\sum_{k=1}^{N_{\text{traj}}}\sum_{j\in\text{bound}}\left(p^*(\overrightarrow{r_{k,j}})\right)^{1/\lambda}} \; then\ as\ N_{\text{traj}} \to \infty \; \frac{E\left[\sum_{i\in\text{unbound}}\left(p^*(\vec{r_i})\right)^{1/\lambda}\right]}{E\left[\sum_{j\in\text{bound}}\left(p^*(\vec{r_j})\right)^{1/\lambda}\right]} \tag{SI-11}$$

Substituting into equation 15 again:

$$\Delta G^\circ = RT\ln\left(\frac{E\left[\sum_{i\in\text{unbound}}\left(p^*(\vec{r_i})\right)^{1/\lambda}\right]}{E\left[\sum_{j\in\text{bound}}\left(p^*(\vec{r_j})\right)^{1/\lambda}\right]}\right) - RT\ln\left(\frac{V_u}{V^o}\right) \tag{SI-12}$$

So as $N_{\text{traj}} \to \infty$ the expected ratio will be equivalent to Eq. 14 no matter the frame capture rate or number of trajectories. Effectively the frame capture rate is like a prefactor to the summation of populations and can be factored out. The sum of states of all trajectories multiplied by $\frac{1}{N_{\text{traj}}}$ effectively creates a sample mean which converges to the true expected value. We show that the number of trajectories is inconsequential to the computed free energy in (**Fig 2C,D,F and SI-3).** Practically speaking though, more trajectories reduces the statistical uncertainty of the computed free energy and frame capture rate must be frequent enough to observe very large ratio differences between states, but not so frequent as to cause disc space concerns or slow-down of MD code.

Note on bin size in reweighting. The bin size utilized should be carefully selected to allow for convergence of all relevant bins in the states described while still accurately estimating the curvature of the energy surface. These two requirements oppose one another: the former requires large bins due to the limits of finite sampling, whereas the latter requires small bins to accurately estimate the curvature by using a Riemann sum estimation of the integral. For the benzamidine-trypsin system, cubic-shaped bin volumes between 3-125 Å^3 provide statistically indiscernible results. This stability over nearly 2 orders of magnitude suggests reweighting bin size will accurately capture population ratios as long as 1) the range is adequate for sufficient sampling and 2) that discretization closely follows the integral. In the unbound state, the free energy surface is approximately flat, so the Riemann sum approximation introduces negligible discretization error at any bin size. In the bound state, the partition function is dominated by the lowest free energy bin(s), whose Boltzmann-weighted populations are exponentially higher than those of higher-energy bins. Consequently, discretization errors in the less-populated bins have a minimal contribution to the state population. Large bin sizes introduce significant error only when they become comparable to the volume over which the free energy surface changes appreciably.

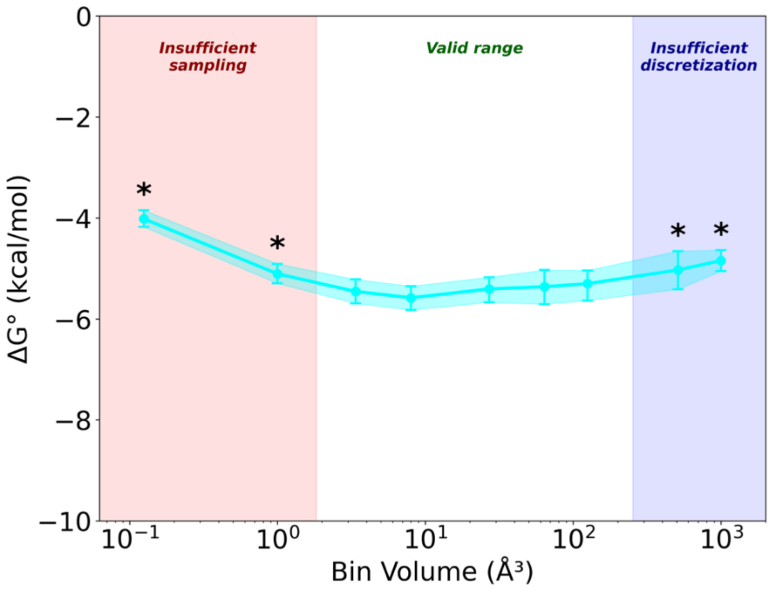

Figure S16 Bin size effect on ΔG^o predicted from trypsin-benzamidine simulations. Cubic bins were varied in size for reweighting of high temperature simulations prior to the calculation of ΔG^o. The bound and unbound state definitions were 5A COM movement from starting position. * denotes statistically significant differences p<0.05.

Note on Markov State Modeling of ΔG^o_{error} contribution from ligand return events. To explore the effect of ligand rebinding on the error ΔG^o_{error}, let us consider a simple Markov state model, which approximates population loss due to the use of absorbing boundaries with volume between them. $q(\vec{r})$ is the probability of state transition as defined in Eq. 6, less than 1. We define three discrete states: bound (B), intermediate (I), and unbound (U), where I is an absorbing state and represents the absorbing boundary **(Fig. S17)**. Let the transition probability from I to B (rebinding to the bound state) be $p = 1 - q(\vec{r})$. Starting from state B, the expected number of times the system visits B before full transition to U follows a geometric distribution with the absorption probability $(1 - p)$. If we denote each visit to B as contributing one unit to the population of the bound state, then the total expected population is:

$$\langle N_B \rangle = \sum_{k=1}^{\infty} k \cdot p^{k-1}(1-p) = \frac{1}{1-p} \qquad \text{(SI-13)}$$

Where k is the index which runs over all visits to state B. $p^{k-1}(1-p)$ is the probability of exactly k total visits to state B (p^{k-1} accounts for k-1 successive rebinding events, and $(1-p)$ accounts for the final transition to state U). For $q(\vec{r})$ = 0.5, this yields $\langle N_B \rangle = 2$. If we place an absorbing boundary at I that terminates trajectories prematurely (i.e., missing all rebinding events from I to B), we measure a population of 1 instead of 2. The resulting free energy error is:

$$\Delta G^o_{error} = -RT \ln\left(\frac{population_{true}}{population_{measured}}\right) = -RT\ln(2) \approx 0.41 \text{ kcal/mol} \qquad \text{(SI-14)}$$

For lower rebinding probabilities (e.g., p = 0.1), the expected population is $\langle N_B \rangle = \frac{1}{1-0.1} = 1.11$ and $\Delta G^o_{error} \approx 0.06$ kcal/mol. As $q(\vec{r}) \to 1, p \to 0$, and $\Delta G^o_{error} \to 0$. We empirically observed this for benzamidine-trypsin in **Fig. 2E**, when the behavior is indicative of low-to-moderate rebinding there is minimal error. If we apply this Markov model to the benzamidine-trypsin simulations, using our observed $q(\vec{r})$ in the bound state simulations (ligand and protein), we can estimate the G^o_{error} based on Eq. SI-13 and SI-14. When we compare this error with the actual observed error introduced as a function of the position of the absorbing boundary with respect to the starting position of the ligand, the Markov state model and the actual simulation agree within statistical error (**Fig. S17A**). (The Markov state model slightly over-predicts the error at smaller distances, most likely due to partial rebinding events.) Thus, we show that at moderate-to-low probability of rebinding ($p \leq 0.5, q(\vec{r}) \geq 0.5$), we minimally contribute error to the final prediction of the standard state binding free energy, and that the level of error may be analytically approximated based on a Markov state model and observed $q(\vec{r})$. We also apply this analysis to the unbound simulation (ligand alone in water). In the unbound simulation, the Markov state model significantly overestimates the ΔG^o_{error} and may serve as an upper limit to the error from utilizing an absorbing boundary. This is because our simple Markov state model does not distinguish partial rebinding events and treats them as complete return to the origin. However, partial rebinding dominates the unbound state because the translational energy surface of the unbound state is flat, driven by random diffusive motion, rather than containing a low energy well at the origin as in the bound state. Nonetheless, the error at our 5 Å absorbing boundaries is minimal for both the bound and unbound simulations and these errors largely cancel in the ratio in Eq. 14.

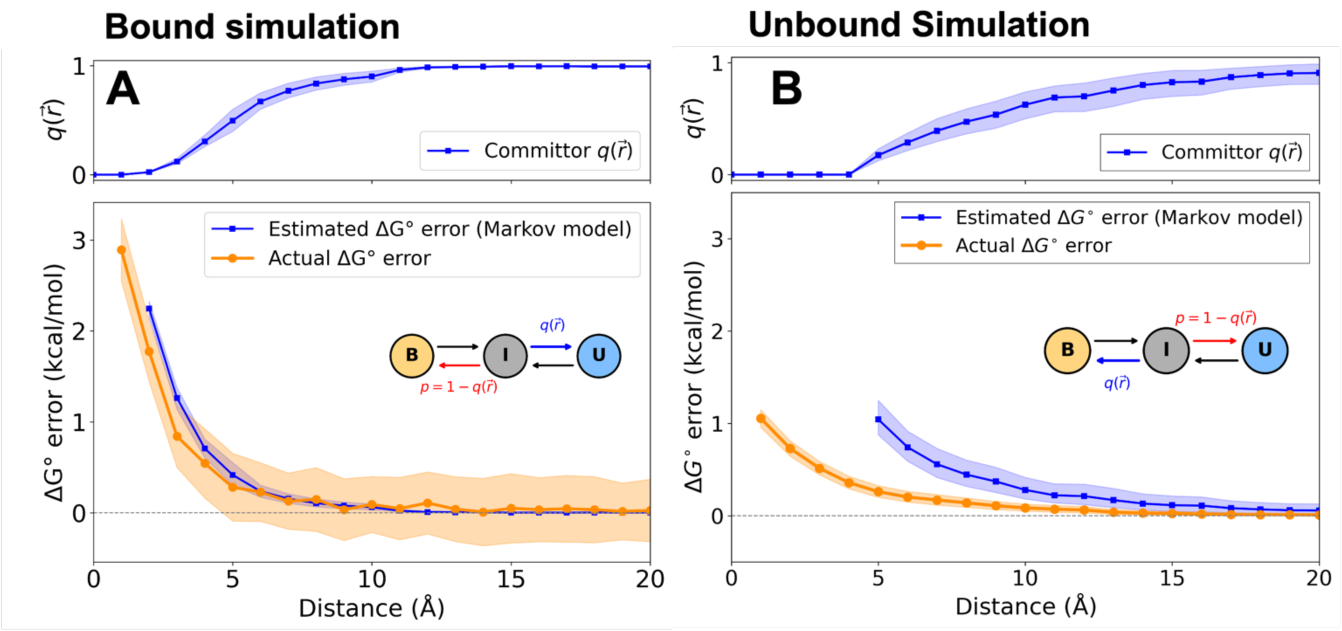

Figure S17. Markov State Modeling of ΔG^{o}_{error} based on $q(\vec{r})$ calculated from trypsin benzamidine simulations. A) Bound state committor function $q(\vec{r})$ for the bound state and the estimated error from the Markov state model, as well as the actual computed ΔG^{o}_{error} in the bound state population based on varying the distance of the absorbing surface from the origin. B) Unbound state committor function $q(\vec{r})$ for the unbound state and the estimated error from the Markov state model, as well as the actual computed ΔG^{o}_{error} in the unbound state population based on varying the distance of the absorbing surface from the origin.

REFERENCES

(1) Chen, W.; Cui, D.; Jerome, S. V.; Michino, M.; Lenselink, E. B.; Huggins, D. J.; Beautrait, A.; Vendome, J.; Abel, R.; Friesner, R. A.; Wang, L. Enhancing Hit Discovery in Virtual Screening through Absolute Protein–Ligand Binding Free-Energy Calculations. *J. Chem. Inf. Model.* **2023**, *63* (10), 3171–3185. https://doi.org/10.1021/acs.jcim.3c00013.

(2) Sinko, W.; Mertz, B.; Shimizu, T.; Takahashi, T.; Terada, Y.; Kimura, S. R. ModBind, a Rapid Simulation-Based Predictor of Ligand Binding and Off-Rates. *J. Chem. Inf. Model.* **2024**. https://doi.org/10.1021/acs.jcim.4c01805.

(3) Friesner, R. A.; Banks, J. L.; Murphy, R. B.; Halgren, T. A.; Klicic, J. J.; Mainz, D. T.; Repasky, M. P.; Knoll, E. H.; Shelley, M.; Perry, J. K.; Shaw, D. E.; Francis, P.; Shenkin, P. S. Glide: A New Approach for Rapid, Accurate Docking and Scoring. 1. Method and Assessment of Docking Accuracy. *J. Med. Chem.* **2004**, *47* (7), 1739–1749. https://doi.org/10.1021/jm0306430.

(4) Sastry, G. M.; Adzhigirey, M.; Day, T.; Annabhimoju, R.; Sherman, W. Protein and Ligand Preparation: Parameters, Protocols, and Influence on Virtual Screening Enrichments. *J. Comput. Aided Mol. Des.* **2013**, *27* (3), 221–234. https://doi.org/10.1007/s10822-013-9644-8.

(5) Halgren, T. A.; Murphy, R. B.; Friesner, R. A.; Beard, H. S.; Frye, L. L.; Pollard, W. T.; Banks, J. L. Glide: A New Approach for Rapid, Accurate Docking and Scoring. 2. Enrichment Factors in Database Screening. *J. Med. Chem.* **2004**, *47* (7), 1750–1759. https://doi.org/10.1021/jm030644s.

(6) Trott, O.; Olson, A. J. AutoDock Vina: Improving the Speed and Accuracy of Docking with a New Scoring Function, Efficient Optimization, and Multithreading. *J. Comput. Chem.* **2010**, *31* (2), 455–461. https://doi.org/10.1002/jcc.21334.

(7) *OpenMM*. https://openmm.org/ (accessed 2023-07-25).

(8) Eastman, P.; Swails, J.; Chodera, J. D.; McGibbon, R. T.; Zhao, Y.; Beauchamp, K. A.; Wang, L.-P.; Simmonett, A. C.; Harrigan, M. P.; Stern, C. D.; Wiewiora, R. P.; Brooks, B. R.; Pande, V. S. OpenMM 7: Rapid Development of High Performance Algorithms for Molecular Dynamics. *PLOS Comput. Biol.* **2017**, *13* (7), e1005659. https://doi.org/10.1371/journal.pcbi.1005659.

(9) AMBER and CHARMM Force Fields for OpenMM, 2023. https://github.com/openmm/openmmforcefields (accessed 2023-07-25).

(10) Wang, J.; Wolf, R. M.; Caldwell, J. W.; Kollman, P. A.; Case, D. A. Development and Testing of a General Amber Force Field. *J. Comput. Chem.* **2004**, *25* (9), 1157–1174. https://doi.org/10.1002/jcc.20035.

(11) Maier, J. A.; Martinez, C.; Kasavajhala, K.; Wickstrom, L.; Hauser, K. E.; Simmerling, C. ff14SB: Improving the Accuracy of Protein Side Chain and Backbone Parameters from ff99SB. *J. Chem. Theory Comput.* **2015**, *11* (8), 3696–3713. https://doi.org/10.1021/acs.jctc.5b00255.

(12) Jorgensen, W. L.; Chandrasekhar, J.; Madura, J. D.; Impey, R. W.; Klein, M. L. Comparison of Simple Potential Functions for Simulating Liquid Water. *J. Chem. Phys.* **1983**, *79* (2), 926–935. https://doi.org/10.1063/1.445869.

(13) James, D. I.; Smith, K. M.; Jordan, A. M.; Fairweather, E. E.; Griffiths, L. A.; Hamilton, N. S.; Hitchin, J. R.; Hutton, C. P.; Jones, S.; Kelly, P.; McGonagle, A. E.; Small, H.; Stowell, A. I. J.; Tucker, J.; Waddell, I. D.; Waszkowycz, B.; Ogilvie, D. J. First-in-Class

Chemical Probes against Poly(ADP-Ribose) Glycohydrolase (PARG) Inhibit DNA Repair with Differential Pharmacology to Olaparib. *ACS Chem. Biol.* **2016**, *11* (11), 3179–3190. https://doi.org/10.1021/acschembio.6b00609.
(14) MCGONAGLE, A. E.; Jordan, A.; Waszkowycz, B.; HUTTON, C.; WADDELL, I.; HITCHIN, J. R.; SMITH, K. M.; HAMILTON, N. M. 2,4-Dioxo-Quinazoline-6-Sulfonamide Derivatives as Inhibitors of Parg. WO2016092326A1, June 16, 2016. https://patents.google.com/patent/WO2016092326A1/en (accessed 2024-05-13).
(15) Li, X.; Shen, C.; Zhu, H.; Yang, Y.; Wang, Q.; Yang, J.; Huang, N. A High-Quality Data Set of Protein–Ligand Binding Interactions Via Comparative Complex Structure Modeling. *J. Chem. Inf. Model.* **2024**, *64* (7), 2454–2466. https://doi.org/10.1021/acs.jcim.3c01170.
(16) Wang, F.; Jeon, K. O.; Salovich, J. M.; Macdonald, J. D.; Alvarado, J.; Gogliotti, R. D.; Phan, J.; Olejniczak, E. T.; Sun, Q.; Wang, S.; Camper, D.; Yuh, J. P.; Shaw, J. G.; Sai, J.; Rossanese, O. W.; Tansey, W. P.; Stauffer, S. R.; Fesik, S. W. Discovery of Potent 2-Aryl-6,7-Dihydro-5H-Pyrrolo[1,2-a]Imidazoles as WDR5-WIN-Site Inhibitors Using Fragment-Based Methods and Structure-Based Design. *J. Med. Chem.* **2018**, *61* (13), 5623–5642. https://doi.org/10.1021/acs.jmedchem.8b00375.
(17) Do, S.; Hu, H.; Kolesnikov, A.; Lee, W.; Tsui, V. H.; Wang, X.; Wen, Z. Pyrazolo[3,4-c]Pyridine Compounds and Methods of Use. US9260425B2, February 16, 2016. https://patents.google.com/patent/US9260425/en (accessed 2026-01-20).
(18) Berghausen, J.; Buschmann, N.; Furet, P.; Gessier, F.; Lisztwan, J. H.; Holzer, P.; Jacoby, E.; Kallen, J.; Masuya, K.; Soldermann, C. P.; REN, H.; Stutz, S. Substituted Isoquinolinones and Quinazolinones. US9051279B2, June 9, 2015. https://patents.google.com/patent/US9051279B2/en?oq=US-9051279-B2 (accessed 2026-01-20).
(19) Cournia, Z.; Chipot, C.; Roux, B.; York, D. M.; Sherman, W. Free Energy Methods in Drug Discovery—Introduction. In *Free Energy Methods in Drug Discovery: Current State and Future Directions*; ACS Symposium Series; American Chemical Society, 2021; Vol. 1397, pp 1–38. https://doi.org/10.1021/bk-2021-1397.ch001.
(20) Loncharich, R. J.; Brooks, B. R.; Pastor, R. W. Langevin Dynamics of Peptides: The Frictional Dependence of Isomerization Rates of N-Acetylalanyl-N′-Methylamide. *Biopolymers* **1992**, *32* (5), 523–535. https://doi.org/10.1002/bip.360320508.
(21) Mollica, L.; Decherchi, S.; Zia, S. R.; Gaspari, R.; Cavalli, A.; Rocchia, W. Kinetics of Protein-Ligand Unbinding via Smoothed Potential Molecular Dynamics Simulations. *Sci. Rep.* **2015**, *5* (1), 11539. https://doi.org/10.1038/srep11539.
(22) Nguyen, H.; Roe, D. R.; Swails, J.; Case, D. A. PYTRAJ v1.0.0.Dev1: Interactive Data Analysis for Molecular Dynamics Simulations, 2016. https://doi.org/10.5281/zenodo.44612.
(23) Roe, D. R.; Cheatham, T. E. I. PTRAJ and CPPTRAJ: Software for Processing and Analysis of Molecular Dynamics Trajectory Data. *J. Chem. Theory Comput.* **2013**, *9* (7), 3084–3095. https://doi.org/10.1021/ct400341p.
(24) Grossfield, A.; Patrone, P. N.; Roe, D. R.; Schultz, A. J.; Siderius, D.; Zuckerman, D. M. Best Practices for Quantification of Uncertainty and Sampling Quality in Molecular Simulations [Article v1.0]. *Living J. Comput. Mol. Sci.* **2019**, *1* (1), 5067–5067. https://doi.org/10.33011/livecoms.1.1.5067.
(25) Hahn, D.; Bayly, C.; Boby, M. L.; Macdonald, H. B.; Chodera, J.; Gapsys, V.; Mey, A.; Mobley, D.; Benito, L. P.; Schindler, C.; Tresadern, G.; Warren, G. Best Practices for Constructing, Preparing, and Evaluating Protein-Ligand Binding Affinity Benchmarks [Article v1.0]. *Living J. Comput. Mol. Sci.* **2022**, *4* (1), 1497–1497. https://doi.org/10.33011/livecoms.4.1.1497.
(26) Edward, J. T. Molecular Volumes and the Stokes-Einstein Equation. *J. Chem. Educ.* **1970**, *47* (4), 261. https://doi.org/10.1021/ed047p261.
(27) Miyamoto, S.; Shimono, K. Molecular Modeling to Estimate the Diffusion Coefficients of Drugs and Other Small Molecules. *Molecules* **2020**, *25* (22), 5340. https://doi.org/10.3390/molecules25225340.
